# Knowledge structure, latent topics, and emerging trends in quercetin-immunity research from 2006 to 2025: a bibliometric and LDA topic-modeling analysis

**DOI:** 10.3389/fnut.2026.1932000

**Published:** 2026-09-09

**Authors:** Congcong Qin, Weiwei Wang, Yuqing Li, Zhengyun Tian, Kailiang Fan, Qinyuan Du, Tejin Ba, Shuanglin Zhang, Guochen Li, Li Kong

**Affiliations:** 1 Institute of Chinese Medical Literature and Culture, Shandong University of Traditional Chinese Medicine, Jinan, Shandong, China; 2 Affiliated Hospital of Shandong University of Traditional Chinese Medicine, Jinan, Shandong, China; 3 The First Clinical Medical College, Shandong University of Traditional Chinese Medicine, Jinan, Shandong, China; 4 Emergency and Critical Care Medicine Center, Shandong University of Traditional Chinese Medicine, Jinan, Shandong, China; 5 Inner Mongolia International Mongolian Hospital, Hohhot, Inner Mongolia, China

**Keywords:** bibliometrics, immunity, inflammation, latent Dirichlet allocation topic modeling, oxidative stress, quercetin, systems pharmacology

## Abstract

**Objective:**

Quercetin is a representative flavonol at the interface of natural-product pharmacology, nutrition, and immune regulation, with reported anti-inflammatory, antioxidant, antiviral, antiallergic, antitumor, and metabolic activities. This systematic mapping review aimed to characterize the publication trajectory, knowledge structure, latent themes, and emerging frontiers of quercetin-immunity research from 2006 to 2025.

**Methods:**

English-language original articles and reviews published from January 1, 2006 to December 31, 2025 were retrieved from the Web of Science Core Collection, Scopus, and PubMed. Following harmonized eligibility reassessment, field standardization, and cross-database deduplication, bibliometric analysis, science mapping, keyword co-occurrence, temporal and burst analyses, thematic maturity assessment, citation and co-citation analyses, and latent Dirichlet allocation (LDA) topic modeling were integrated to examine the explicit and latent knowledge structures. Mapping-level risk of bias was qualitatively assessed.

**Results:**

The searches yielded 15,938 raw records, of which 10,619 unique publications were included, comprising 7,754 original articles and 2,865 reviews. Of these, 6,440 publications appeared during 2021–2025, accounting for 60.65% of the dataset and demonstrating rapid growth. The field spanned natural products, pharmacology, molecular biology, nutrition, and immunology, with China, India, and the United States as the leading contributors. Stable mechanistic terms included oxidative stress, inflammation, signal transduction, apoptosis, NF-κB, TNF, IL-6, and IL-1β, indicating a knowledge core centered on inflammatory cytokine networks, redox homeostasis, transcriptional regulation, and cell-fate control. LDA resolved 12 topics spanning inflammatory signaling, systems pharmacology, senescence, oxidative organ injury, tumor immunity, neuroinflammation and ferroptosis, infection and intestinal inflammation, allergy and mast-cell activation, delivery systems, and bioavailability. Recent frontiers included molecular docking, network pharmacology, protein–protein interaction, Kyoto Encyclopedia of Genes and Genomes (KEGG) and Gene Ontology analyses, ferroptosis, macrophage polarization, and nuclear factor erythroid 2-related factor 2 (Nrf2) signaling, indicating a shift toward multi-target, systems-level mechanistic investigation. The mapping-level assessment indicated low-to-moderate residual risk.

**Conclusion:**

Quercetin-immunity research has evolved from broad anti-inflammatory studies into an interdisciplinary field focused on inflammatory signaling, oxidative stress, immune-cell function, disease-specific immunopathology, and systems pharmacology. Future studies should prioritize physiologically attainable exposures, active metabolites, immune-cell specificity, disease-stage effects, multi-omics validation, and clinically relevant dosing.

## 1 Introduction

Quercetin is one of the most widely distributed and extensively investigated flavonols in the plant kingdom, and it represents a prototypical small-molecule bioactive compound at the interface of natural product pharmacology and nutritional immunology (1). The term quercetin can be traced to the Latin word quercetum, reflecting its historical association with early isolation from plants of the genus Quercus. In the early twentieth century, Stanisław Kostanecki and colleagues conducted a series of studies on the synthetic chemistry and structural elucidation of flavonoids, which helped transform quercetin from a crude component of plant pigments into an independent chemical entity with a defined structure (2). Subsequently, Albert Szent-Györgyi’s work on vitamin P and dietary flavonoids broadened the understanding of the biological functions of plant polyphenols (3). With the development of antioxidant biology, cardiovascular protection, dietary polyphenol research, and natural product pharmacology, quercetin gradually became a central research object linking phytochemistry, nutritional intervention, and disease-mechanism investigation (4).

Quercetin is widely present in onion (*Allium cepa* L.), apple (*Malus domestica* Borkh.), berries, tea (*Camellia sinensis* (L.) Kuntze), grape (*Vitis vinifera* L.), buckwheat (*Fagopyrum esculentum* Moench), and a variety of medicinal and edible plants (1, 5). Chemically, quercetin is 3,3′,4′,5,7-pentahydroxyflavone (1). Its multiple phenolic hydroxyl groups and conjugated aromatic ring system provide a structural basis for free-radical scavenging, metal-ion chelation, redox homeostasis regulation, and interaction with protein targets (6, 7). In “Quercetin, Inflammation and Immunity”, Li et al. systematically summarized the physicochemical properties, dietary sources, absorption and metabolism, bioavailability characteristics, and regulatory effects of quercetin on inflammatory responses and immune function (8). Their work emphasized that the biological activity of quercetin should not be interpreted from a single pharmacodynamic perspective, but should be evaluated within an integrated framework involving inflammation, oxidative stress, and immune regulation.

The immune system mediates pathogen clearance, supports tissue repair, and maintains organismal homeostasis (9). Sustained or excessive immune activation, however, contributes to chronic inflammation, autoimmune injury, infection-related immunopathology, metabolic inflammation, neuroinflammation, and remodeling of the tumor immune microenvironment (10). Early studies generally described the relationship between quercetin and the immune system in terms of anti-inflammatory and antioxidant activity (8). With advances in molecular immunology and cell biology, research has progressively moved toward the molecular regulation of pro-inflammatory cytokine release, immune-cell activation, transcription-factor signaling, and regulated cell-death pathways. In a lipopolysaccharide (LPS)-induced RAW 264.7 macrophage model, Sung-Yeon Cho et al. reported that quercetin inhibited the production of pro-inflammatory cytokines, downregulated nitric oxide (NO) and inducible nitric oxide synthase (iNOS), and modulated extracellular signal-regulated kinase (ERK), p38 mitogen-activated protein kinase (p38 MAPK), and nuclear factor kappa B (NF-κB)/IκB signaling (11).

Subsequent studies have expanded the mechanistic scope of quercetin-mediated immune regulation. Pattern-recognition receptors, mitogen-activated protein kinase (MAPK), NF-κB, nuclear factor erythroid 2-related factor 2 (Nrf2), heme oxygenase-1 (HO-1), inflammasomes, and pyroptosis have gradually emerged as major research directions in this field. Sun et al. showed in BV-2 microglial cells that quercetin attenuated LPS-induced inflammatory responses and enhanced Nrf2/HO-1-mediated antioxidant defense (12). Domiciano et al. further reported that quercetin inhibited activation of the NLR family pyrin domain-containing 3 (NLRP3) and absent in melanoma 2 (AIM2) inflammasomes by interfering with apoptosis-associated speck-like protein containing a caspase recruitment domain (ASC) oligomerization, thereby reducing interleukin-1β (IL-1β) release (13). In a collagen-induced arthritis model, Yang et al. found that quercetin restored the balance between T helper 17 (Th17) cells and regulatory T (Treg) cells, while suppressing the expression of NLRP3, caspase-1, IL-1β, tumor necrosis factor (TNF), interleukin-6 (IL-6), cyclooxygenase-2 (COX-2), and iNOS (14). Weng et al. further showed in a human mast-cell model that quercetin inhibited cytokine release and exerted stronger inhibitory effects than cromolyn on several inflammatory markers (15). These findings suggest that the functional scope of quercetin has extended beyond macrophage-associated inflammatory regulation to allergic responses and mast-cell activation.

The disease contexts in which quercetin has been investigated continue to expand. Current studies have examined its effects in autoimmune diseases, inflammatory bowel disease, viral infection, lung injury, metabolic inflammation, neuroinflammation, tumor-associated immune regulation, and aging-related inflammation. Clinical investigations of dasatinib plus quercetin introduced quercetin into the research framework of senolytics, senescent-cell clearance, and chronic inflammatory remodeling (16, 17). In intestinal inflammation, quercetin has been linked to gut microbiota homeostasis, mucosal barrier integrity, NLRP3 inflammasome activation, and epithelial immune homeostasis (18–20). In neuroinflammation, its regulatory effects have been further associated with microglial activation, the Nrf2/HO-1 pathway, mitochondrial injury, oxidative lipid damage, and ferroptosis (12, 21, 22). Collectively, these advances indicate that quercetin-related immunological research has shifted from early assessments of anti-inflammatory activity to mechanistic investigations of the targets, pathways, and regulatory networks through which quercetin acts in distinct disease settings and immune-cell microenvironments.

Despite this progress, the existing literature remains highly fragmented. First, most experimental studies focus on a single disease model, cell type, or signaling pathway, limiting the ability to define the overall knowledge structure and directional transition of the field. Second, although mechanistic reviews have summarized the anti-inflammatory, antioxidant, antiviral, and immunomodulatory effects of quercetin, they are generally not designed to quantify the growth rates of different research themes, identify core knowledge anchors, characterize contribution patterns, or describe stage-specific evolution. Third, quercetin research spans natural product chemistry, nutrition, pharmacology, molecular immunology, disease biology, and systems pharmacology, with relevant publications dispersed across different databases and journal systems. An integrative mapping of the field therefore remains necessary. In addition, quercetin is characterized by low aqueous solubility, complex metabolic transformation, and limited bioavailability. The concentrations used *in vitro* often differ substantially from achievable *in vivo* exposure levels, and its clinical translational value requires reassessment within a more complete evidence framework (23–25).

Bibliometric analysis provides a systematic macro-level characterization of publication growth, database and journal coverage, country/region and institutional contributions, collaboration patterns, citation foundations, and keyword networks (26). Keyword co-occurrence analysis identifies explicitly indexed concepts and their relational structure (27). Latent Dirichlet allocation (LDA) extends this explicit analysis by inferring latent topics and disease contexts from term distributions and co-occurrence patterns in titles, abstracts, and indexing terms (28, 29). Building on this methodological complementarity, we included English-language original articles and reviews on quercetin and immunity indexed in the Web of Science Core Collection, Scopus, and PubMed between 2006 and 2025. Within a systematic mapping framework, bibliometric analysis, science mapping, and LDA topic modeling were integrated to examine publication and collaboration patterns, citation and co-citation structures, temporal keyword evolution, burst topics, thematic maturity, and latent semantic topics. By linking explicit conceptual networks with latent semantic structures, this study aimed to delineate the field’s knowledge architecture and developmental trajectory. It also sought to identify emerging directions characterized by a shift from descriptions of anti-inflammatory effects toward multi-target, systems-level mechanistic investigation. The resulting map provides a reproducible bibliographic evidence base for subsequent mechanistic validation, disease-specific research, and translational application.

## 2 Materials and methods

### 2.1 Study design and analytical framework

This study integrated multi-database bibliometric analysis, science mapping, and latent Dirichlet allocation (LDA) topic modeling to systematically characterize the knowledge structure, latent topics, and evolving frontiers of quercetin-immunity research from 2006 to 2025 (26, 28). Bibliometric analysis was used to quantify publication output, growth phases, journal distribution, contribution patterns, collaboration network structure, and citation foundations (26). Keyword co-occurrence analysis, temporal stratification, burst-term detection, and thematic maturity assessment were further applied to delineate the explicit knowledge structure of the field and its stage-specific evolution (27). LDA topic modeling was used to identify latent research themes from the semantic information embedded in titles, abstracts, and keywords (28). All analyses were conducted on the same cross-database deduplicated dataset, thereby ensuring a consistent data foundation across descriptive bibliometric statistics, science-mapping analyses, and topic modeling.

### 2.2 Data sources, search strategy, and eligibility criteria

Bibliographic records were retrieved from three major databases: Web of Science Core Collection (WoSCC), Scopus, and PubMed. All searches were conducted and the records were exported on 4 July 2026. The core search query was defined as follows: (quercetin OR quercetol) AND immun*. The WoSCC search was restricted to the Topic field, the Scopus search to the Article title, Abstract, and Keywords fields, and the PubMed search to the Title/Abstract field. The publication period was limited to January 1, 2006 through December 31, 2025. Eligible document types were restricted to Article and Review, and the language was restricted to English. The initial exports from WoSCC, Scopus, and PubMed yielded 3,680, 9,743, and 2,515 records, respectively. After harmonized eligibility reassessment, 3,598, 9,743, and 2,457 eligible source records were retained from the three databases, respectively. The record counts before and after cross-database integration, together with the search fields and eligibility criteria, are summarized in Table 1. Full details of database-specific search fields, screening restrictions, and re-screened records are provided in Supplementary Table 1. Eligibility reassessment and secondary screening were independently performed by two researchers (CQ and WW) using the prespecified publication-year, language, and document-type criteria; record-level discrepancies were resolved by consensus with reference to the source-database records and eligibility criteria.

**Table 1:** Search strategy, eligibility criteria, and dataset construction.

Database	Search field	Core search concept	Time span	Document type	Language	Raw records	Eligible records
Web of Science Core Collection	Topic	(quercetin OR quercetol) AND immun*	2006–2025	Article; Review	English	3,680	3,598
Scopus	Article title; Abstract; Keywords	(quercetin OR quercetol) AND immun*	2006–2025	Article; Review	English	9,743	9,743
PubMed	Title/Abstract	(quercetin OR quercetol) AND immun*	2006–2025	Article; Review	English	2,515	2,457

### 2.3 Record parsing, field standardization, and cross-database deduplication

The exported records were re-parsed using a predefined local processing workflow and were subjected to secondary screening according to publication year, language, and document type. Fields requiring standardization included title, author names, journal title, publication year, digital object identifier (DOI), PubMed ID, abstract, author keywords, database-indexed keywords, Medical Subject Headings (MeSH), author addresses, institutions, countries/regions, and citation counts. Cross-database deduplication used normalized DOI as the primary matching identifier and PubMed ID as an exact auxiliary identifier. For records lacking both DOI and PubMed ID, normalized title combined with the surname of the first author was used as a fallback matching strategy. Title normalization involved case harmonization, punctuation cleaning, whitespace normalization, and removal of common articles to reduce matching errors caused by formatting differences. Across the three databases, 15,938 raw records were retrieved; 15,798 source records remained after re-screening, and 10,619 unique publications were finally obtained after cross-database deduplication.

### 2.4 Bibliometric indicators and counting strategies

#### 2.4.1 Descriptive bibliometric and collaboration indicators

Descriptive indicators comprised annual publication output, document-type composition, database-source overlap, leading journals, productive authors, and contributions from countries/regions and institutions. Annual output was summarized by publication year and across three analytical periods: 2006–2015, 2016–2020, and 2021–2025. Country/region and institutional outputs were derived from author-affiliation addresses using full counting, whereby each publication contributed one credit to every unique country/region and institution represented. Because multinational or multi-institutional publications could contribute more than one credit, the resulting shares were non-additive. Author-level output was calculated after name standardization. Collaboration was characterized by the number of authors per publication, the number and proportion of multinational publications, and temporal changes in these indicators. For records duplicated across databases, the highest available citation count was retained for descriptive citation analyses.

Corpus-composition variability was evaluated using a publication-level nonparametric bootstrap comprising 20,000 resamples of the 10,619 unique publications (random seed = 20,260,818). Two-sided 95% percentile bootstrap intervals were estimated for annual, country/region, and institutional publication counts. Year-by-document-type strata were resampled jointly, whereas country/region and institutional affiliations retained their original full-counting assignments within each resampled publication. These intervals quantify the stability of corpus-derived counts under empirical resampling; they do not account for uncertainty arising from database coverage or indexing practices.

#### 2.4.2 Country-level counting sensitivity analysis

Country-level output was recalculated using fractional counting to quantify inflation attributable to international coauthorship. For a publication involving m unique countries/regions, each represented country/region received a weight of 1/m, ensuring that the total country-level credit assigned to each publication equalled one; single-country publications retained a weight of one. Records without mapped country/region information were excluded from this comparison. Robustness across counting methods was assessed using total assigned credits, the full-to-fractional credit ratio and corresponding percentage inflation, country-specific rank changes, Spearman rank correlation, Kendall’s tau-b, and overlap among the top five, top 10, and top 20 countries/regions. Full counting was retained for the primary descriptive analysis, whereas fractional counting served as a sensitivity analysis of country-level output and ranking stability.

### 2.5 Keyword standardization and science-mapping analysis

Keywords were obtained from author keywords, database-indexed keywords, and PubMed MeSH terms. Term standardization included case harmonization, singular-plural merging, abbreviation standardization, synonym merging, and removal of low-information indexing terms. Orthographic, symbolic, and indexing variants were harmonized to TNF (TNF-α, TNFa, TNF-alpha, TNFα, and tumor necrosis factor), Nrf2 (Nrf-2, NRF2, NFE2L2, and transcription factor Nrf2), and NF-κB (NF-kappa B, NFκB, and nuclear factor kappa B). The complete raw-to-canonical mapping dictionary, comprising all merged variants, canonical labels, and excluded terms, is provided in Supplementary Table 2. Morphological variants such as flavonoid/flavonoids, antioxidant/antioxidants, and plant extract/plant extracts were merged into unified terms. Keyword co-occurrence was quantified by full binary counting after within-publication deduplication. For standardized keyword i in publication $kX_{\mathrm{ik}}=1$ if the keyword was present and 0 otherwise. Keyword frequency and pairwise co-occurrence strength were defined as $f_{i}=\sum_{k}X_{\mathrm{ik}}$ and $C_{\mathrm{ij}}=\sum_{k}X_{\mathrm{ik}}\;X_{\mathrm{jk}}$ , respectively. Network visualization was restricted to nodes with $f_{i}\geq 1,150$ and edges with $C_{\mathrm{ij}}\geq 600$ . Normalized association strength was calculated using the Salton cosine, $S_{\mathrm{ij}}=\frac{C_{\mathrm{ij}}}{\sqrt{f_{i}\;f_{j}}}$ . The core conceptual structure was then organized according to high-frequency keywords and their co-occurrence relationships, yielding thematic modules related to inflammatory cytokines and signaling regulation, oxidative stress and redox homeostasis, cell fate and signal transduction, natural products and comparative phytochemistry, systems pharmacology, and disease-specific immune mechanisms.

### 2.6 Temporal evolution, burst keywords, and thematic maturity analysis

The study period was stratified into four intervals: 2006–2010, 2011–2015, 2016–2020, and 2021–2025. For each interval, the frequency of standardized keywords, the distribution of thematic modules, and the recent proportion of each theme were calculated. The burst index was defined as the ratio between the observed frequency of a keyword in the target interval plus 1 and the average expected frequency of that keyword across the four intervals plus 1. To reduce the influence of random fluctuations among low-frequency terms, keywords with a total frequency below 20 or an interval-specific frequency below 10 were excluded from burst ranking. Thematic maturity was assessed using three indicators: thematic coverage, an internal density proxy, and recent proportion. Thematic coverage was defined as the number of unique publications containing keywords assigned to a given theme. The internal density proxy was defined as the mean co-occurrence strength between any two keywords within the same theme. Recent proportion was defined as the percentage of keyword frequency in 2021–2025 relative to the total frequency of that theme. Based on these indicators, themes with high coverage and high internal density were classified as motor themes; themes with high coverage but relatively stable recent proportions were classified as basic transverse themes; and themes with high recent proportions and rapidly increasing coverage were classified as emerging themes or methodological frontiers.

### 2.7 Citation and co-citation analyses

Highly cited publications were ranked in descending order according to citation counts in the deduplicated dataset to identify the influential bibliographic foundation of the field. Co-citation analysis was conducted using the cited references field from WoSCC. Cited references were preferentially matched and merged by DOI. For references without DOI information, standardized matching was performed using the first author, publication year, and source journal. For cited reference i and eligible WoSCC source publication k , $R_{\mathrm{ik}}=1$ if publication k cited reference i , and $R_{\mathrm{ik}}=0$ otherwise. Reference citation frequency was defined as $C_{i}=\sum_{k}R_{\mathrm{ik}}$ , andpairwise co-citation strength as $C_{\mathrm{ij}}=\sum_{k}R_{\mathrm{ik}}R_{\mathrm{jk}}$ . References with $C_{i}\geq 20$ and links with $C_{\mathrm{ij}}\geq 5$ were retained in the operational network. These thresholds were selected through sensitivity analyses across $C_{i}$ = 10, 15, 20, 25, 30, and 50 and $C_{\mathrm{ij}}$ = 3, 5, and 10, balancing preservation of the connected core against excessive network density. Salton cosine similarity, $S_{\mathrm{ij}}=\frac{C_{\mathrm{ij}}}{\sqrt{C_{i}C_{j}}}$ , was calculated as a normalized association measure. Reference themes were assigned according to title, source journal, and keyword information, and were grouped into knowledge modules including quercetin/flavonoid/polyphenol pharmacology, oxidative stress and redox biology, inflammation and immune signaling, inflammasomes and pyroptosis, aging and chronic immune remodeling, and network pharmacology and computational mechanisms.

### 2.8 LDA topic modeling and latent semantic topic identification

The corpus for LDA topic modeling was constructed by combining publication titles, abstracts, author keywords, database-indexed keywords, and MeSH terms, covering all 10,619 included publications. Text preprocessing included conversion to lowercase, standardization of Greek letters and special symbols, punctuation and number cleaning, removal of search terms, removal of domain-specific stop words, filtering of low-information generic terms, and standardization of mechanistic phrases. Before modeling, phrases such as oxidative stress, NF-κB, Nrf2, molecular docking, network pharmacology, protein–protein interaction, gut microbiota, tumor microenvironment, and reactive oxygen species were integrated as fixed terms to avoid semantic fragmentation and term dilution. The final document-term matrix contained 3,500 terms.

The LDA topic model was fitted using online variational Bayes estimation (28). Candidate topic numbers were set as K = 6, 8, 10, 12, and 14. Model selection was based on four criteria calculated from a random sample of 5,000 publications: normalized pointwise mutual information (NPMI) coherence, log perplexity, topic redundancy, and manual interpretability (29, 30). Topic redundancy was represented by the mean Jaccard similarity between sets of high-weight terms across different topics. K = 12 provided better thematic interpretability with lower interpretive cost and was therefore selected as the final topic number (Supplementary Figure 2). The final model was fitted using all 10,619 publications. Each topic was assigned a descriptive label by jointly evaluating its 20 highest-weight terms, the publications with the highest topic proportions, and stage-specific topic weights. The average topic weight of each topic across the four time intervals and its recent proportion during 2021–2025 were then calculated.

### 2.9 Reproducibility and bias control

All analyses were conducted using locally exported raw bibliographic records and a predefined analytical pipeline. The workflow covered database export, harmonized eligibility re-screening, field standardization, cross-database deduplication, descriptive statistics, keyword merging, temporal stratification, burst detection, co-citation analysis, thematic maturity assessment, and LDA topic modeling. Data from three databases were integrated to reduce coverage bias associated with a single database. Text preprocessing rules, topic-number selection procedures, model parameters, random seeds, and output formats were fixed to ensure traceability and reproducibility.

Metrics at the country and regional, institutional, and author levels, together with citation based analyses, were influenced by database coverage, the structure and completeness of affiliation information, the consistency of author name standardization, and the availability of citation fields. These results should therefore be interpreted as bibliometric indicators derived from the available metadata rather than as exhaustive measures of research performance. Keyword analysis and latent Dirichlet allocation topic modeling were based on bibliographic text and database indexing information. Topic labels were treated as domain-informed interpretations of model-derived term weights, representative publications, and temporal distributions rather than as independently observed categories.

## 3 Results

### 3.1 Literature screening and dataset construction

**Figure 1:**
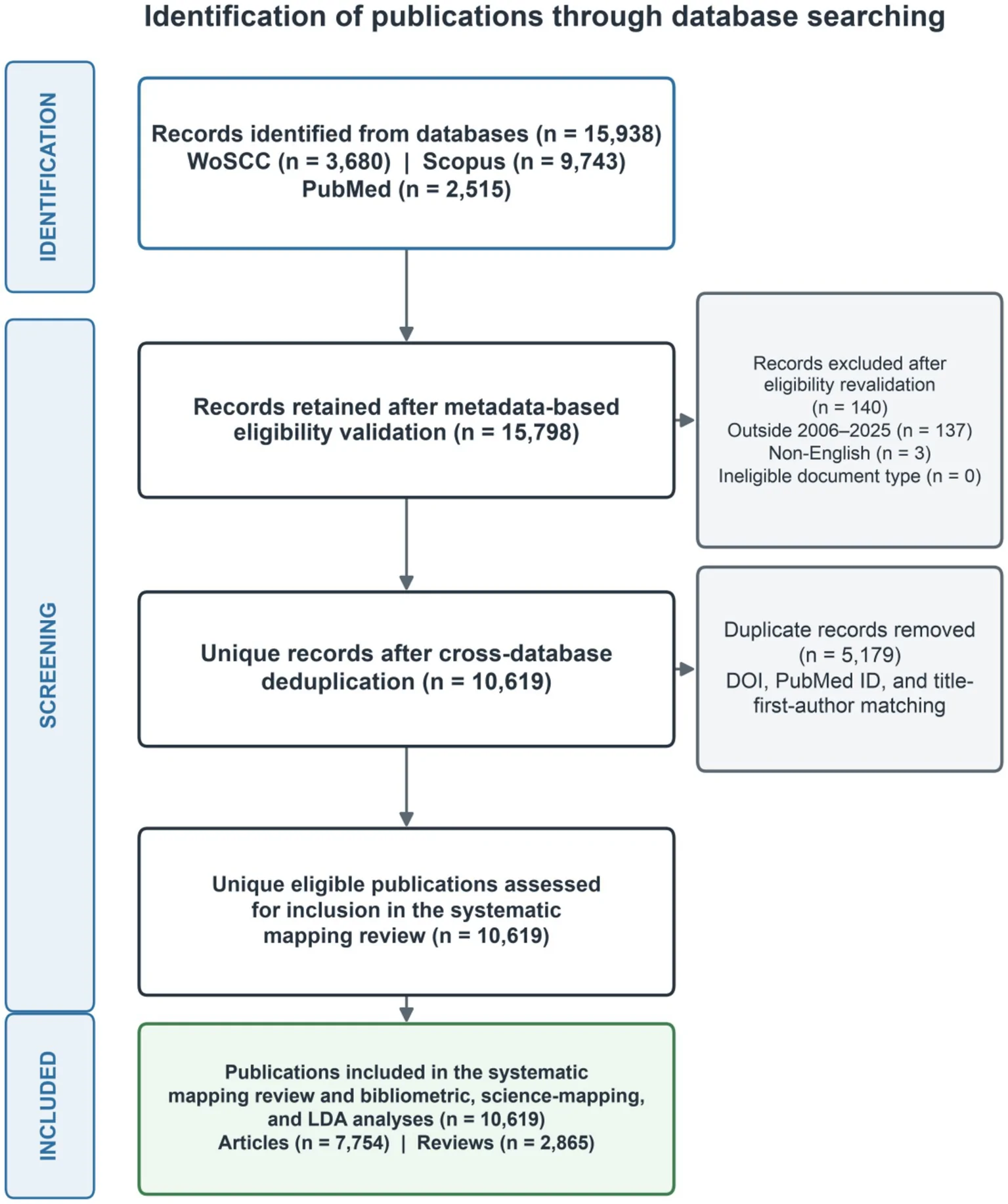
Study selection and dataset construction. Records from WoSCC, Scopus, and PubMed were screened and deduplicated, yielding 10,619 publications for bibliometric, science-mapping, and LDA analyses.

The three database searches yielded 15,938 raw records, including 3,680 from the Web of Science Core Collection (WoSCC), 9,743 from Scopus, and 2,515 from PubMed. After harmonized reassessment based on publication year, language, and document type, 15,798 source records were retained. Cross database deduplication was then performed using normalized digital object identifier (DOI), PubMed ID, and a fallback matching strategy based on title and first author. A total of 10,619 unique publications were finally included, comprising 7,754 original articles and 2,865 reviews. This dataset served as the unified data foundation for the bibliometric analysis, science mapping, and latent Dirichlet allocation (LDA) topic modeling in this study (Figure 1).

Database coverage analysis showed that, among the 10,619 unique publications, 7,706 (72.57%) were indexed exclusively in one database, 661 (6.22%) were shared by exactly two databases, and 2,252 (21.21%) were indexed in all three databases—Web of Science Core Collection (WoSCC), Scopus, and PubMed. Scopus contributed the largest number of database-exclusive records (*n* = 6,883; 64.82%), followed by WoSCC (*n* = 769; 7.24%) and PubMed (*n* = 54; 0.51%). The pairwise overlaps comprised 512 publications shared by WoSCC and Scopus (4.82%), 87 shared by Scopus and PubMed (0.82%), and 62 shared by WoSCC and PubMed (0.58%). These findings demonstrate substantial heterogeneity in database coverage and support the integration of all three databases to construct a more comprehensive systematic mapping corpus (Supplementary Figure 1).

### 3.2 Annual publication trends and expansion of collaboration

**Figure 2:**
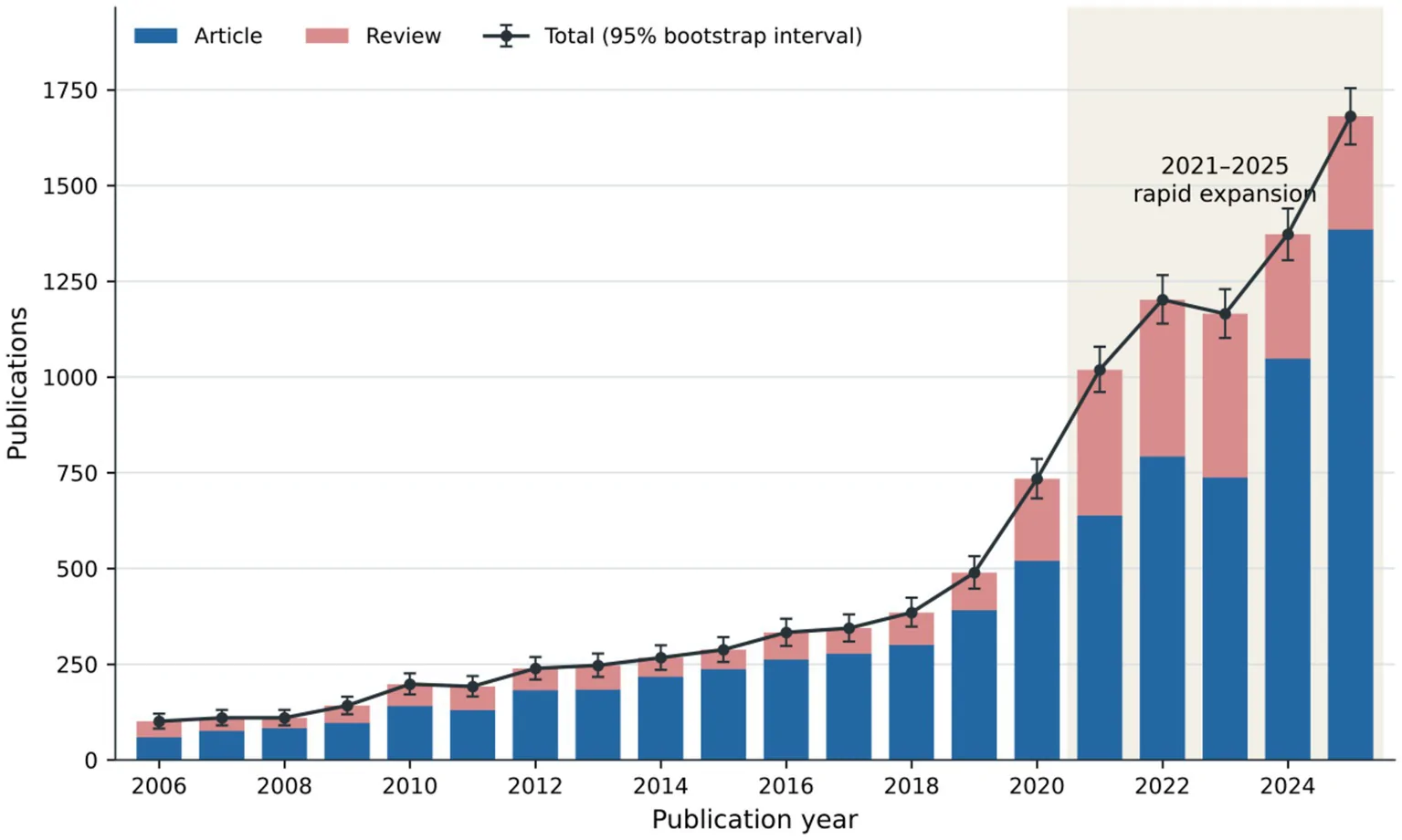
Annual publication output and document-type distribution from 2006 to 2025. Error bars represent two-sided 95% percentile intervals derived from 20,000 publication-level bootstrap resamples.

From 2006 to 2025, the annual output of quercetin and immunity related research increased continuously. The number of publications rose from 101 in 2006 to 1,681 in 2025, representing an approximately 16.6 fold increase. When examined by growth phase, the period from 2006 to 2015 represented a slow accumulation stage, with 1,894 publications, accounting for 17.84% of the total dataset. The period from 2016 to 2020 represented a stable growth stage, with 2,285 publications, accounting for 21.52%. The field then entered a rapid expansion stage from 2021 to 2025, during which 6,440 publications were published, accounting for 60.65% of all included records (Figure 2). In the most recent five year period, 4,600 publications were classified as Article and 1,840 as Review, indicating concurrent growth in original research output and review based knowledge synthesis (Supplementary Table 3A). Publication output was 101 in 2006 (95% bootstrap interval, 82–121), 1,019 in 2021 (961–1,079), and 1,681 in 2025 (1,607–1,754), indicating that the marked temporal expansion was robust to empirical corpus resampling.

The collaboration structure also changed over time. The mean number of authors per publication increased from 5.08 during 2006 to 2010 to 6.97 during 2021 to 2025. A total of 2,231 publications involved multinational collaboration, accounting for 21.01% of all included records. This proportion increased from 17.10% during 2006 to 2010 to 22.28% during 2016 to 2020, and remained at 21.24% during 2021 to 2025. The median citation count of recent publications was lower than that of earlier publications, which is consistent with the shorter citation accumulation period of newly published literature. The concurrent increase in publication volume, team size, and international collaboration indicates that the field has moved from an early stage of relatively dispersed exploration to a phase of rapid expansion involving multiple research teams (Supplementary Table 3B).

### 3.3 Geographic, institutional, and author contributions

#### 3.3.1 Country-level output and counting-method sensitivity

Under full counting, country/region output was concentrated in Asia. China ranked first with 3,877 publications (36.51% of the corpus), followed by India with 1,299, the United States with 1,160, South Korea with 573, and Italy with 472. Because multinational publications were credited once to each participating country/region, these counts are non-additive.

**Figure 3:**
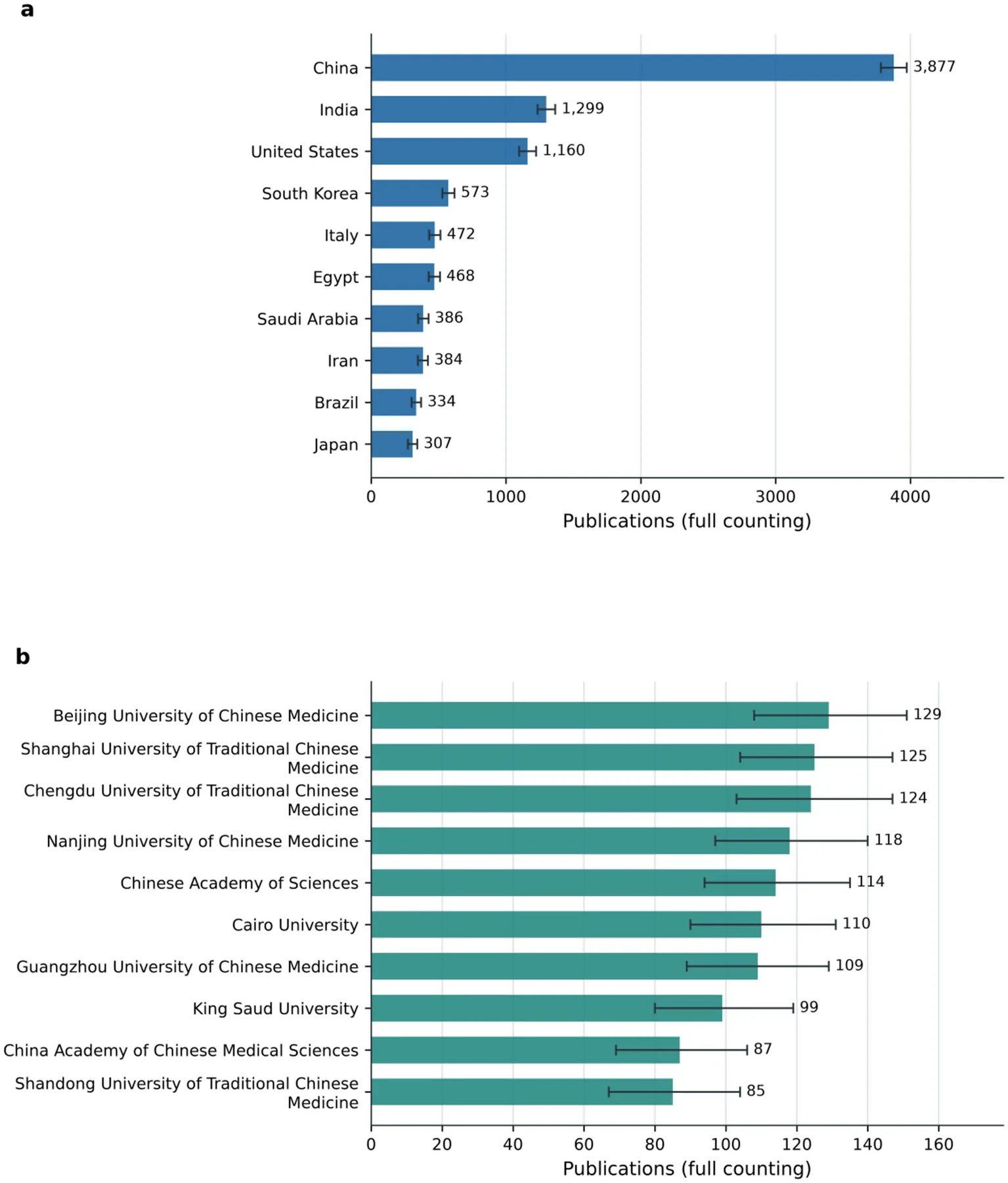
Country/region and institutional contribution landscape based on full counting. **(a)** Top 10 countries/regions by publication output. **(b)** Top 10 institutions by publication output. Error bars represent two-sided 95% percentile intervals derived from 20,000 publication-level bootstrap resamples. Fractional-count sensitivity estimates for country/region output are provided in Supplementary Table 7.

Country information was available for 10,535 of the 10,619 included publications, of which 2,231 (21.18%) involved two or more countries/regions. Full counting yielded 13,811 country credits, compared with 10,535 under fractional counting, representing 3,276 excess credits and an aggregate inflation of 31.10%. Country rankings remained highly concordant across methods (Spearman ρ = 0.9904; Kendall τ_b_ = 0.9346): the five leading countries were unchanged, nine of the top 10 overlapped, and all top 20 were retained. Saudi Arabia showed the largest shift among the full-count top 10, moving from seventh to eleventh under fractional counting. Thus, full counting inflated absolute output, particularly for highly collaborative countries, but had limited influence on the leading country hierarchy (Figure 3; Supplementary Tables 4, 7).

#### 3.3.2 Institutional and author contributions

Leading institutions included Beijing University of Chinese Medicine, Shanghai University of Traditional Chinese Medicine, Chengdu University of Traditional Chinese Medicine, Nanjing University of Chinese Medicine, and the Chinese Academy of Sciences. The prominence of universities of traditional Chinese medicine, natural-product research institutions, and basic-life-science organizations reflects the disciplinary foundations of quercetin-immunity research (Figure 3; Supplementary Table 4).

Author-level output was weakly concentrated. Cho, Jae Youl contributed 38 publications, whereas Wang, Wei and Zhang, Yi each contributed 34. The 20 most productive standardized author entries collectively accounted for 491 publications (4.62% of the corpus), indicating that research output was distributed across numerous investigators and research teams rather than concentrated among a small group of authors (Supplementary Table 4).

### 3.4 Journal distribution and interdisciplinary characteristics

The included publications were distributed across journals from multiple disciplines, reflecting the intersection of natural products, pharmacology, molecular biology, nutrition, and immunology. The most productive journal was Journal of Ethnopharmacology, with 415 publications, accounting for 3.91% of the dataset, followed by Frontiers in Pharmacology with 285 publications, International Journal of Molecular Sciences with 265, Phytomedicine with 192, and Nutrients with 145. The top 10 journals collectively published 1,878 articles, representing 17.69% of all included publications.

**Figure 4:**
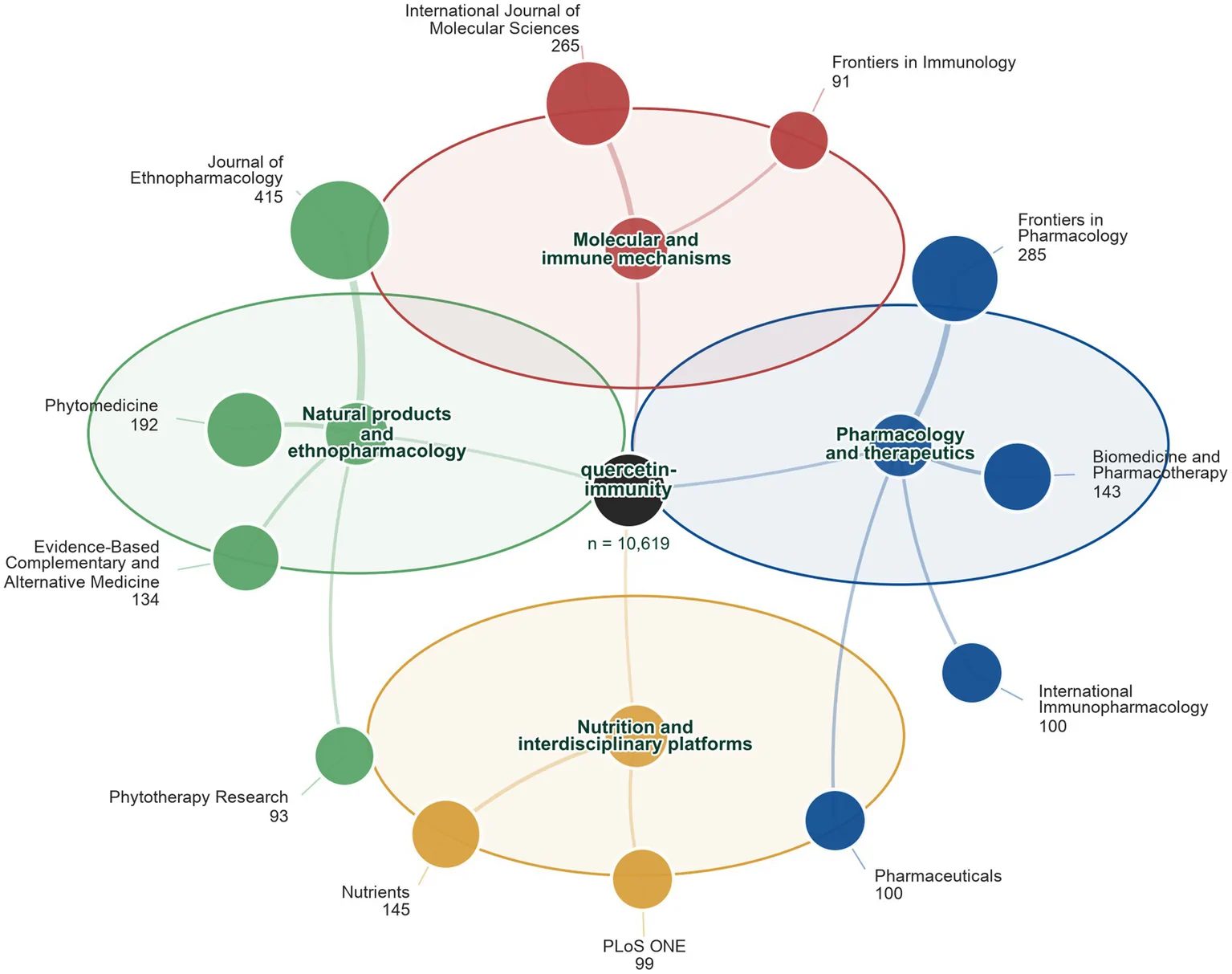
Source-journal landscape and disciplinary positioning of high-output journals. Node size represents publication output, and colors indicate disciplinary clusters.

The high output of Journal of Ethnopharmacology and Phytomedicine reflects the close connection between this field and ethnopharmacology, botanical medicine, and natural bioactive compounds. The substantial contributions from Frontiers in Pharmacology, International Journal of Molecular Sciences, and International Immunopharmacology further indicate that the research focus has extended toward molecular mechanisms, signal transduction, and immune regulation. Overall, the journal distribution suggests that quercetin immunity research is not confined to a single pharmacological or nutritional domain, but has developed into an interdisciplinary dissemination structure grounded in natural products and centered on immune and inflammatory mechanisms (Figure 4).

### 3.5 Highly cited publications and the co-citation knowledge base

Citation count analysis included 10,565 publications with available citation information, representing 99.49% of the dataset; among these, 10,027 publications had been cited at least once. Highly cited publications were concentrated in oxidative stress, inflammatory responses, flavonoid pharmacology, inflammasomes, immunosenescence, and reviews on quercetin and immunity. The most cited publication was “Free radicals, metals and antioxidants in oxidative stress-induced cancer” by Valko et al., published in Chemico-Biological Interactions, with 5,809 citations (31). “Quercetin, Inflammation and Immunity” by Li et al., published in Nutrients, received 1,825 citations (8). “Targeting the NLRP3 inflammasome in inflammatory diseases” by Mangan et al., published in Nature Reviews Drug Discovery, received 1,567 citations (32). The senescent cell clearance study involving dasatinib plus quercetin by Hickson et al. was cited 1,138 times, indicating that quercetin related research has entered the context of immunosenescence and chronic inflammation intervention (17).

**Figure 5:**
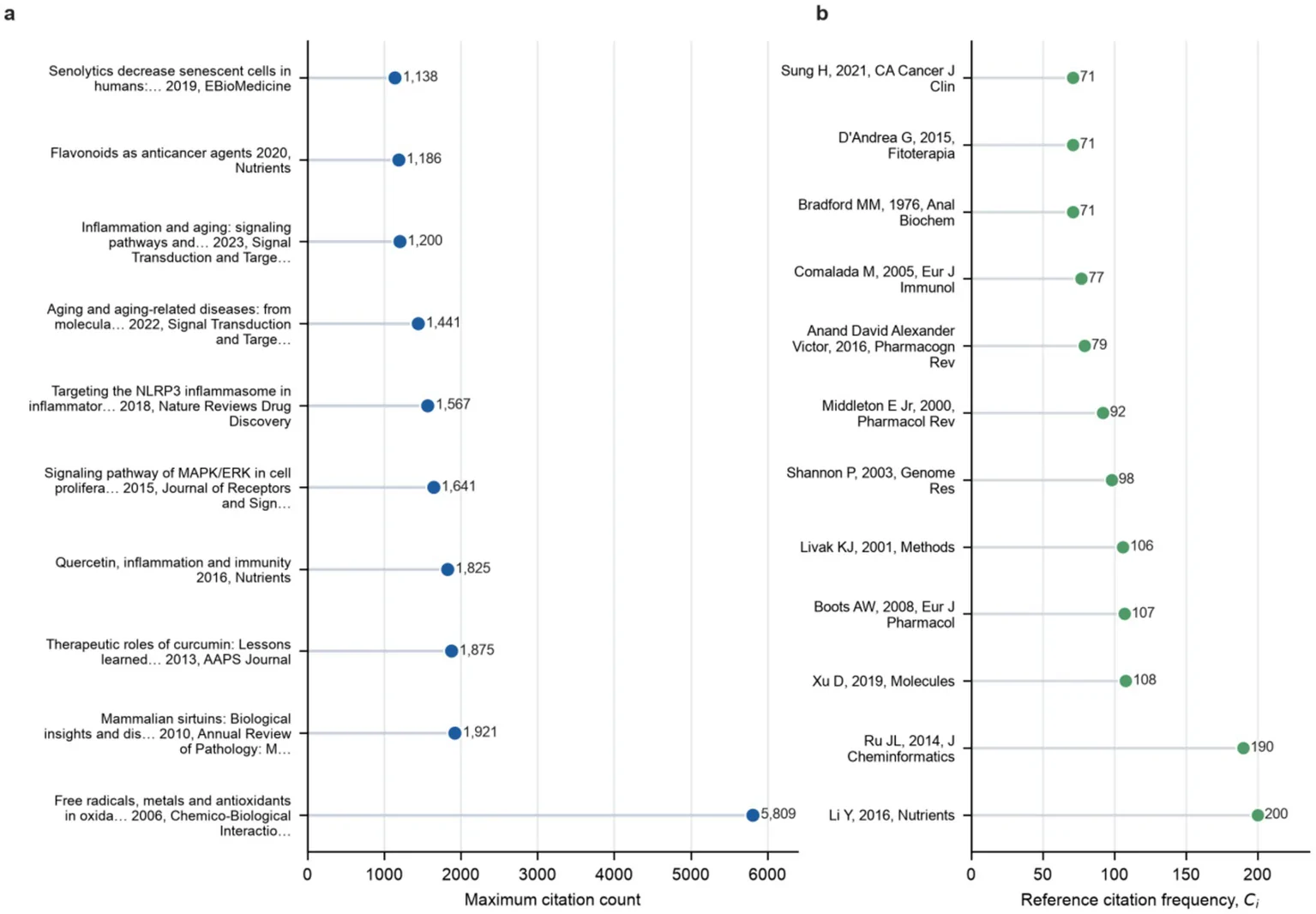
Citation and co-citation knowledge base. **(a)** Highly cited publications in the deduplicated three-database corpus. **(b)** The 12 highest-frequency cited-reference nodes in the eligible WoSCC subset; all ties at rank 10 were retained. Reference citation frequency *Ci* denotes the number of source publications citing reference i. The operational co-citation network retained references with *Ci* ≥ 20 and links with *C*_ij_ ≥ 5.

Co-citation analysis was restricted to 3,598 eligible WoSCC source publications, of which 3,596 contained cited-reference data, yielding 232,648 raw cited-reference entries and 174,848 normalized unique references. Application of the $C_{i}\geq 20$ threshold retained 241 reference nodes, whereas application of $C_{\mathrm{ij}}\geq 5$ retained 822 co-citation links; the largest connected component comprised 216 nodes, corresponding to 89.63% of the retained network. The most frequently cited retained references were the review by Li et al. (2016) in Nutrients ( $C_{i}=200$ ), the TCMSP article by Ru et al. (2014) in Journal of Cheminformatics ( $C_{i}=190$ ), the study by Xu et al. (2019) in Molecules ( $C_{i}=108$ ), and the review by Boots et al. (2008) in European Journal of Pharmacology ( $C_{i}=107$ ) (6–8, 33). Prominent co-citation links connected Shannon et al. (2003) with Ru et al. (2014) ( $C_{\mathrm{ij}}=72$ ), Szklarczyk et al. (2023) with Ru et al. (2014) ( $C_{\mathrm{ij}}=52$ ), and Xu et al. (2019) with Li et al. (2016) ( $C_{\mathrm{ij}}=33$ ) (6, 8, 33–35). The resulting structure linked quercetin, flavonoid and polyphenol pharmacology with network pharmacology, molecular docking and protein-interaction methodology (Figure 5; Supplementary Table 8) (33–36).

### 3.6 Keyword co-occurrence analysis delineates the explicit conceptual structure

**Figure 6:**
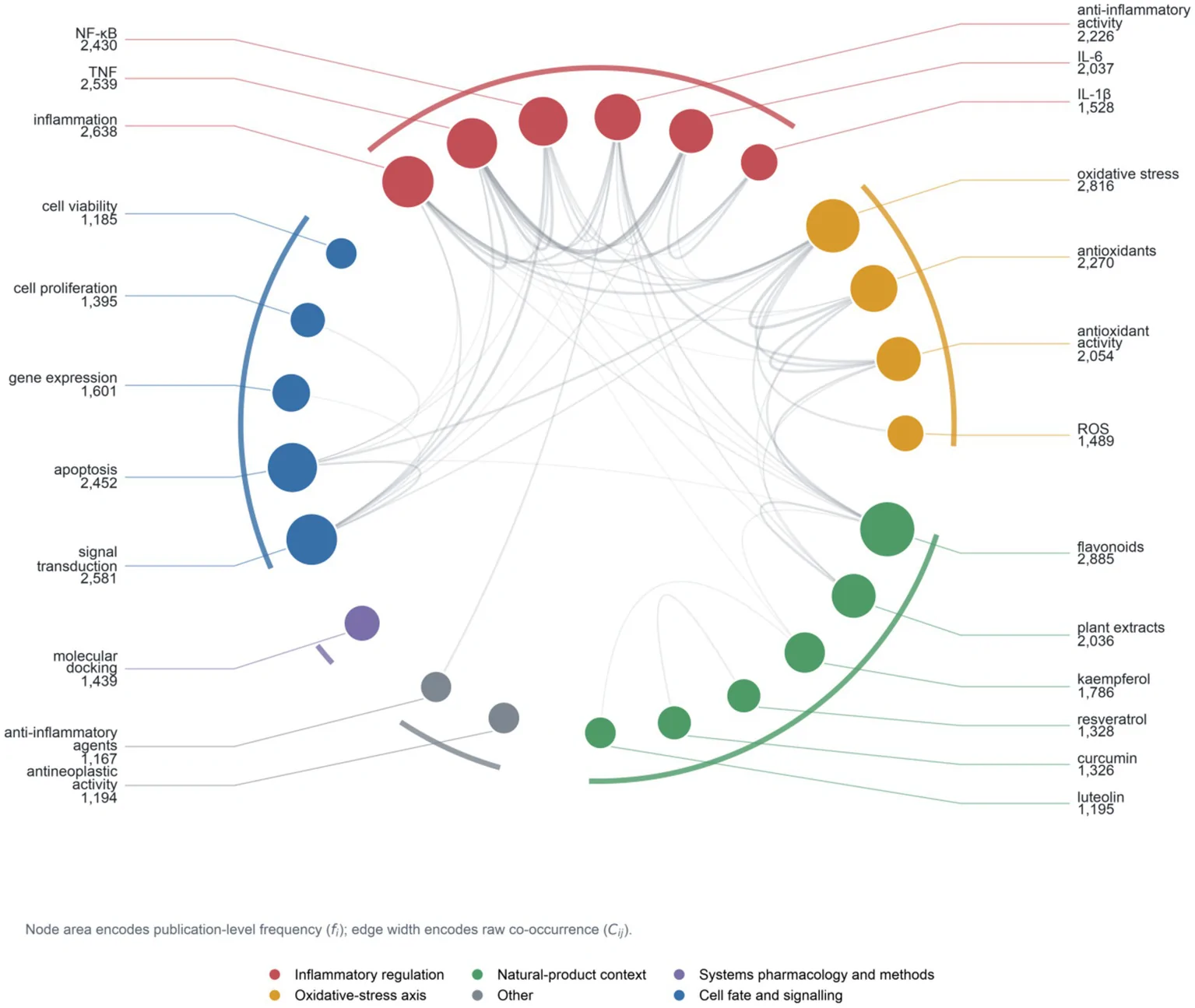
Keyword co-occurrence and conceptual structure. Node area is proportional to publication-level keyword frequency (*f*ᵢ), and edge width represents raw pairwise co-occurrence (*C*ᵢⱼ). The network retains keywords with *f*ᵢ ≥ 1,150 and edges with *C*ᵢⱼ ≥ 600.

Keyword co occurrence analysis showed that the explicit conceptual structure of quercetin and immunity research was primarily organized around natural flavonoids, oxidative stress, inflammatory regulation, signal transduction, and cell fate. The highest publication-level keyword frequencies were observed for flavonoids (*n* = 2,885), oxidative stress (*n* = 2,816), inflammation (*n* = 2,638), signal transduction (*n* = 2,581), TNF (*n* = 2,539), apoptosis (*n* = 2,452), NF-κB (*n* = 2,430), antioxidants (*n* = 2,270), anti-inflammatory activity (*n* = 2,226), and antioxidant activity (*n* = 2,054) (Figure 6; Supplementary Table 5A).

From the perspective of thematic structure, the high frequency keywords could be organized into several interconnected mechanistic axes. The inflammatory regulation axis comprised inflammation, TNF, IL-6, IL-1β, NF-κB, and anti inflammatory activity. The oxidative stress axis comprised oxidative stress, ROS, antioxidants, and antioxidant activity. The cell fate and signal transduction axis comprised apoptosis, signal transduction, gene expression, and cell proliferation. The natural product comparison axis comprised flavonoids, kaempferol, curcumin, resveratrol, and luteolin. This structure indicates that quercetin and immunity research is not defined by a single anti inflammatory narrative, but instead forms a multilayered mechanistic framework centered on redox homeostasis, pro inflammatory cytokine networks, transcription factor regulation, and changes in cell fate.

### 3.7 LDA topic modeling identifies latent semantic topics

**Figure 7:**
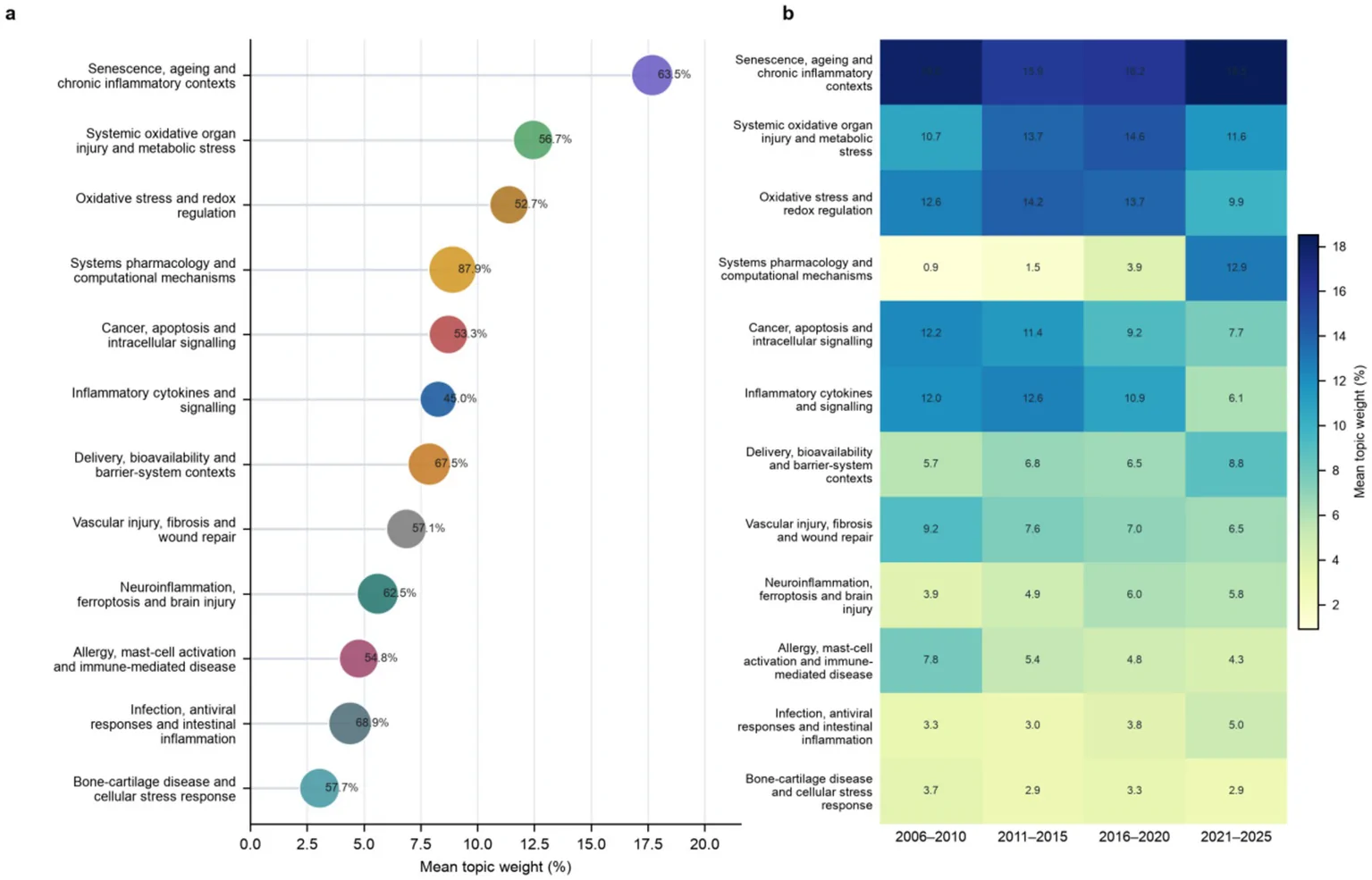
LDA-derived latent topics and temporal prevalence. **(a)** Mean topic weights of the 12 LDA topics. **(b)** Topic prevalence across four time periods from 2006 to 2025.

Latent Dirichlet allocation (LDA) topic modeling included all 10,619 publications. The corpus was constructed from titles, abstracts, author keywords, database indexed keywords, and Medical Subject Headings (MeSH) terms. After comparison of candidate topic numbers, K = 12 was selected for the final model. LDA identified 12 latent semantic topics, covering inflammatory cytokines and signaling, systems pharmacology and computational mechanisms, senescence, aging and chronic inflammatory contexts, systemic oxidative organ injury and metabolic stress, cancer, apoptosis and intracellular signaling, bone and cartilage disease and cellular stress response, vascular injury, fibrosis and wound repair, oxidative stress and redox regulation, neuroinflammation, ferroptosis and brain injury, allergy, mast cell activation and immune mediated disease, infection, antiviral responses and intestinal inflammation, and delivery, bioavailability and barrier system contexts (Figure 7; Supplementary Tables 5B, 6).

The latent Dirichlet allocation (LDA) results complemented the keyword co occurrence structure. Terms such as inflammatory cytokines, NF-κB, mitogen activated protein kinase (MAPK), TNF, iNOS, and COX-2 supported the topic of inflammatory cytokines and signaling. Terms including flavonoids, antioxidant, anti inflammatory, phenolic, phytochemical, and antioxidant activity corresponded to the topic of oxidative stress and redox regulation. Network pharmacology, molecular docking, Kyoto Encyclopedia of Genes and Genomes (KEGG), enrichment, PI3K, Akt, and TNF were concentrated in the topic of systems pharmacology and computational mechanisms. Compared with keyword co occurrence analysis, LDA further separated disease related and translational contexts, including aging related chronic inflammation, neuroinflammation and ferroptosis, infection and antiviral responses, delivery and bioavailability, bone and cartilage diseases, and vascular fibrosis. These results indicate that latent semantic topics provide a more refined classification of research contexts than high frequency keywords alone.

### 3.8 Temporal evolution of explicit and latent themes

Temporal stratified keyword analysis showed a transition from descriptions of natural product activity toward mechanistic analysis of immune regulatory networks. During 2006 to 2010, high frequency themes mainly included flavonoids, antioxidants, NF-κB, inflammation, antioxidant activity, apoptosis, and anti inflammatory activity, indicating that early research focused primarily on flavonoid natural products, antioxidant activity, and anti inflammatory effects. During 2011 to 2015, NF-κB, signal transduction, plant extracts, apoptosis, oxidative stress, and TNF became more prominent, suggesting a gradual shift toward inflammatory signaling and cellular regulation. During 2016 to 2020, oxidative stress, NF-κB, signal transduction, inflammation, TNF, and IL-6 ranked among the leading terms, indicating that classical inflammatory cytokine networks and oxidative stress mechanisms constituted the dominant framework of this stage. During 2021–2025, oxidative stress occurred in 1,852 publications, inflammation in 1,706, TNF in 1,729, IL-6 in 1,516, and molecular docking in 1,311 publications.

**Figure 8:**
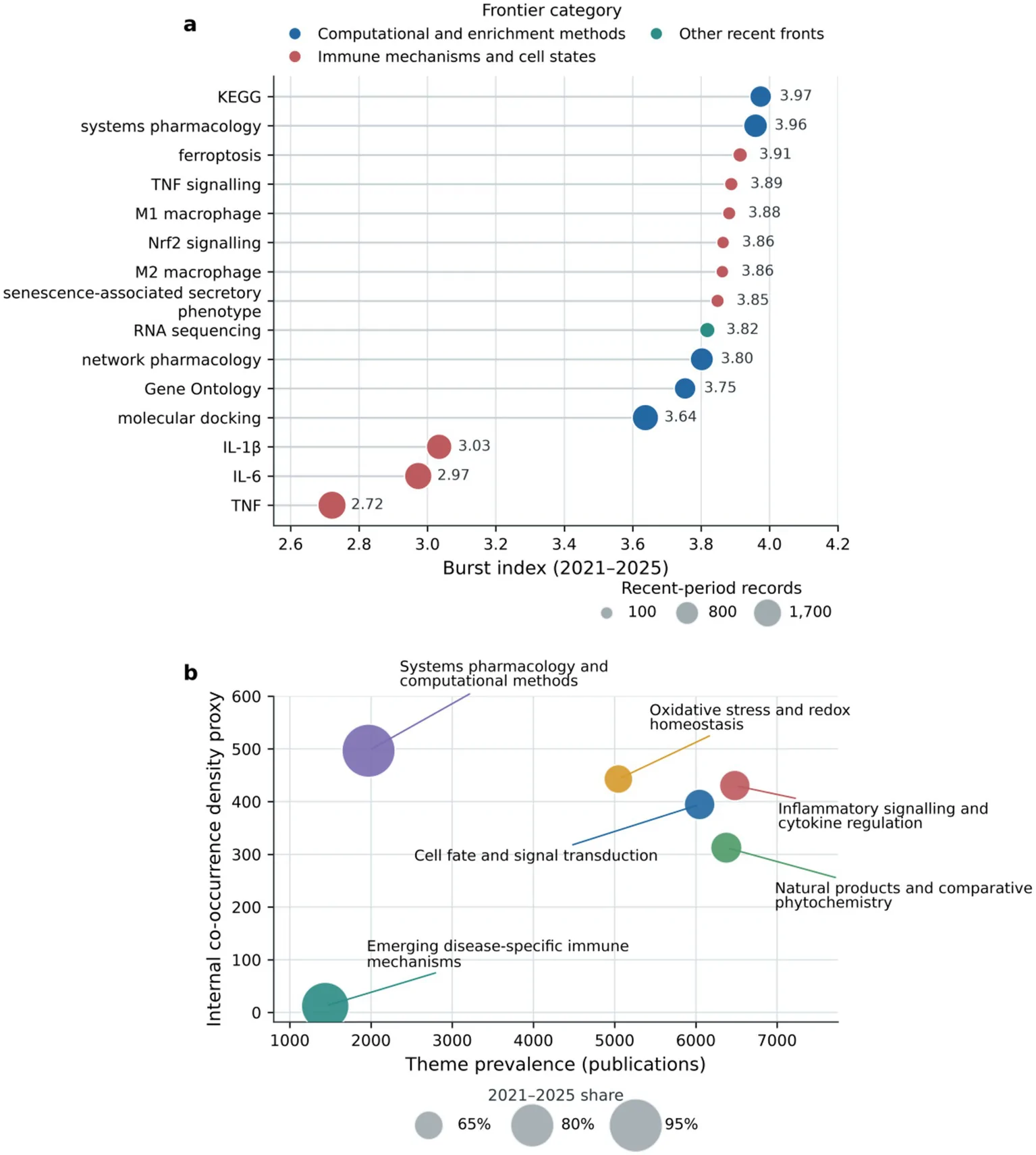
Recent keyword bursts and thematic maturity. **(a)** Descriptive burst indices for selected keywords during 2021–2025; dot area encodes the recent-period publication count. **(b)** Theme prevalence and internal co-occurrence density; bubble area encodes the proportion of theme-keyword occurrences during 2021–2025.

Burst keyword analysis further indicated that 2021 to 2025 was a period of rapid frontier reorganization. During 2021–2025, KEGG occurred 613 times, representing 99.8% of its total frequency; the corresponding values were 873 (99.3%) for systems pharmacology, 788 (95.4%) for network pharmacology, 607 (94.3%) for Gene Ontology, 1,311 (91.1%) for molecular docking, and 714 (88.5%) for protein–protein interaction (Figure 8a; Supplementary Table 5C). Within the mechanistic frontier, ferroptosis, TNF signaling, M1 macrophage, M2 macrophage, Nrf2 signaling, senescence associated secretory phenotype, RNA sequencing, and neuroinflammatory diseases were concentrated predominantly in the most recent five year period.

Stage specific LDA topic weights were consistent with the burst keyword results. The recent proportion of systems pharmacology and computational mechanisms during 2021 to 2025 reached 87.9%, the highest among all LDA topics. The corresponding proportions were 68.9% for infection, antiviral responses and intestinal inflammation; 67.5% for delivery, bioavailability and barrier system contexts; 63.5% for senescence, aging and chronic inflammatory contexts; and 62.5% for neuroinflammation, ferroptosis and brain injury. By contrast, inflammatory cytokines and signaling and oxidative stress and redox regulation persisted throughout the entire study period, suggesting that these represent more mature foundational mechanism topics. Together, explicit keywords and LDA derived latent topics indicate that recent research frontiers have expanded from classical validation of anti inflammatory and antioxidant effects toward systems pharmacology, disease specific immune networks, neuroinflammation, infection immunity, and translational delivery related directions.

### 3.9 Thematic maturity and stratification of the knowledge structure

Thematic maturity analysis delineated six knowledge modules. Inflammatory signaling and cytokine regulation encompassed 6,480 publications and constituted the principal motor theme. Natural products and comparative phytochemistry encompassed 6,375 publications and represented a basic and transversal theme. Cell fate and signal transduction and oxidative stress and redox homeostasis encompassed 6,046 and 5,046 publications, respectively. Collectively, these modules defined the core mechanistic structure of quercetin-immunity research.

Systems pharmacology and computational methods encompassed 1,970 publications, 94.2% of which were published during 2021–2025, identifying this module as the most rapidly expanding methodological frontier. Emerging disease-specific immune mechanisms encompassed 1,436 publications, with 84.7% concentrated in the same period. This module comprised inflammasomes, pyroptosis, ferroptosis, the senescence-associated secretory phenotype, neuroinflammation, and macrophage polarization. Integration with the LDA results resolved the field into four strata: a foundational layer of natural products and flavonoid research; a core mechanistic layer of inflammation and oxidative stress; a methodological frontier of systems pharmacology and computational approaches; and a disease-specific immune-network and translational layer (Figure 8b; Supplementary Table 5D).

### 3.10 Integrated evidence mapping indicates a shift from mechanistic validation to systems level interpretation

Multiple layers of evidence jointly delineated the evolution of quercetin immunity research. Publication trends indicated that the field entered a phase of rapid expansion during 2021 to 2025. Journal and institutional distributions showed that the field is rooted in the intersection of natural product pharmacology, traditional Chinese medicine research, molecular biology, and immune regulation. Highly cited and co citation results indicated that oxidative stress, inflammatory signaling, flavonoid pharmacology, inflammasomes, and systems pharmacology tools constitute the main knowledge base. Keyword co occurrence analysis identified inflammatory cytokines, oxidative stress, signal transduction, and cell fate as the explicit core structure. LDA topic modeling further resolved latent semantic themes related to aging, neuroinflammation, infection immunity, delivery systems, and bioavailability.

A coherent evidence chain was formed across explicit keywords, burst terms, thematic maturity, and recent proportions of LDA topics. Classical themes remained supported by mechanistic terms such as NF-κB, TNF, IL-6, IL-1β, oxidative stress, ROS, and apoptosis. Recent frontiers were concentrated in network pharmacology, molecular docking, systems pharmacology, KEGG, Gene Ontology, protein–protein interaction, ferroptosis, macrophage polarization, senescence-associated secretory phenotype, and neuroinflammation. Taken together, these findings suggest that quercetin immunity research is moving beyond descriptions of isolated anti-inflammatory or antioxidant effects toward multi-target, multi-pathway, disease-specific, and systems level mechanistic interpretation.

### 3.11 Risk-of-bias assessment

The mapping-level assessment indicated low-to-moderate residual risk across the evaluated domains. The integration of three complementary databases, identifier-based deduplication, standardized metadata harmonization, fixed preprocessing and modeling parameters, and multi-criteria LDA model selection strengthened the robustness and reproducibility of the analysis. The remaining variation primarily reflected inherent differences in database indexing, metadata availability, name and affiliation formats, and citation accumulation time. The convergence of findings across publication, citation, keyword, thematic, and topic-modeling analyses supported the stability of the principal field-level patterns.

## 4 Discussion

### 4.1 Principal findings and the two layer thematic map

The 10,619 publications obtained after three database deduplication indicate that quercetin and immunity research has developed a knowledge structure jointly supported by explicit keywords and latent semantic topics. Keyword co occurrence analysis captured the core concepts actively indexed by authors, mainly including oxidative stress, inflammation, signal transduction, apoptosis, NF-κB, TNF, IL-6, and IL-1β. LDA topic modeling further revealed how these concepts are combined across disease contexts, cell types, computational approaches, and translational issues. The former reflects explicitly named research concepts, whereas the latter captures the semantic contexts embedded in the literature. When considered together, the relationship between quercetin and immunity is no longer limited to broad labels such as anti-inflammatory or antioxidant activity, but appears as a multilayered connection among chemical structure, metabolic exposure, inflammatory signaling, immune-cell state, and disease microenvironment.

The co citation evidence was consistent with this structure. “Quercetin, Inflammation and Immunity” by Yao Li et al., published in Nutrients, linked the physicochemical properties, sources, absorption, metabolism, bioavailability, and inflammatory and immunological effects of quercetin (8). The present evidence map shows that the inflammation and immunity framework summarized in that review was continuously refined between 2006 and 2025. Early studies mainly focused on inflammatory cytokines and antioxidant effects, whereas subsequent work extended toward NF-κB, MAPK, Nrf2, inflammasomes, pyroptosis, macrophage polarization, Th17/Treg balance, network pharmacology, and molecular docking. LDA topics further mapped these mechanisms onto contexts such as aging, neuroinflammation, infection, intestinal inflammation, barrier systems, and delivery optimization, suggesting that growth in this field has been driven by both mechanistic deepening and expansion of application scenarios.

### 4.2 Chemical structure, metabolic exposure, and immune effects

The immunopharmacological activity of quercetin is closely related to its flavonol structure (1). The polyphenolic hydroxyl groups and conjugated aromatic rings of 3,3′,4′,5,7-pentahydroxyflavone provide a chemical basis for electron transfer, free-radical scavenging, metal-ion chelation, and interaction with protein targets (6, 7). These structural features help explain why oxidative stress and antioxidants have consistently ranked among the most frequent keywords, and why pathways such as Nrf2/HO-1, ROS/NF-κB, and NOX2/ROS/NF-κB have repeatedly appeared in recent studies.

Immune effects should also be interpreted in relation to *in vivo* exposure. In natural sources, quercetin is often present in glycoside forms and undergoes intestinal hydrolysis, absorption, enterohepatic metabolism, glucuronidation, sulfation, and methylation after intake (23, 24). Detectable circulating forms are often conjugated metabolites rather than high concentrations of free parent compound (24, 25). *In vitro* studies frequently use quercetin concentrations that exceed levels attainable through dietary exposure (25). Mechanistic interpretation therefore needs to consider the parent compound, metabolites, local tissue concentrations, and route of administration. The emergence of delivery, bioavailability and barrier system contexts as an independent LDA topic indicates that translational limitations have become a research object in their own right rather than a peripheral background issue.

### 4.3 Core immune mechanisms represented by explicit keywords

#### 4.3.1 Inflammatory cytokines and the toll-like receptor, MAPK and NF-κB axis

The high frequency of tumor necrosis factor (TNF), interleukin-6 (IL-6), interleukin-1β (IL-1β), cyclooxygenase-2 (COX-2), and inducible nitric oxide synthase (iNOS) indicates that the pro-inflammatory cytokine network represents one of the most stable core mechanisms in quercetin-mediated immune regulation. In RAW 264.7 macrophages, Sung Yeon Cho et al. reported that quercetin inhibited lipopolysaccharide (LPS) induced pro-inflammatory cytokines, nitric oxide (NO), and iNOS expression, while reducing activation of extracellular signal-regulated kinase (ERK), p38 mitogen-activated protein kinase (p38 MAPK), and NF-κB/IκB signaling (11). Endale et al. further interpreted the upstream regulation of innate immune signaling by quercetin through Toll-like receptor 4 (TLR4)/myeloid differentiation primary response 88 (MyD88)/phosphoinositide 3-kinase (PI3K) complex formation, MAPK/activator protein 1 (AP-1), and IκB kinase (IKK)/NF-κB cascades (37). Collectively, quercetin may regulate the full process of downstream pattern recognition receptor signaling, transcription factor activation, and inflammatory mediator production (38, 39). This regulatory pattern applies to multiple immune related cell types, including macrophages, epithelial cells, endothelial cells, and microglia, and helps explain why this mechanism has been repeatedly examined in models of enteritis, arthritis, lung injury, neuroinflammation, and metabolic inflammation.

#### 4.3.2 Coupling of oxidative stress, Nrf2/HO-1, and inflammatory signaling

The persistent prominence of oxidative stress among high frequency keywords suggests that redox homeostasis is a key link between the chemical properties of quercetin and its immunomodulatory effects. In BV-2 microglial cells, Grace Y. Sun et al. found that quercetin exerted a stronger inhibitory effect on LPS induced NO production than cyanidin and promoted Nrf2 mediated expression of heme oxygenase-1 (HO-1) (12). Luo et al. reported that quercetin inhibited nicotinamide adenine dinucleotide phosphate oxidase (NADPH oxidase) derived oxidative stress by inducing HO-1 expression (40). Ok-Joo Sul and Seung Won Ra further showed that quercetin attenuated LPS induced oxidative stress and inflammatory responses through the NOX2/ROS/NF-κB axis (41). Reactive oxygen species (ROS) act both as amplifiers of inflammatory signaling and as key signaling molecules regulating NF-κB, inflammasomes, and cell death pathways (32, 42). The regulation of multiple axes, including Nrf2/HO-1, NOX2/ROS/NF-κB, AMP-activated protein kinase (AMPK), and PI3K/Akt, indicates that the immunomodulatory effect of quercetin is expressed through the coupling of inflammatory suppression and redox homeostasis remodeling.

#### 4.3.3 Inflammasomes, pyroptosis, and the IL-1β axis

IL-1β has maintained high research activity in recent years, consistent with the growing prominence of inflammasome and pyroptosis research. A study by Talita P. Domiciano et al. published in Scientific Reports showed that quercetin inhibited NLRP3 and AIM2 inflammasome mediated IL-1β release and blocked inflammasome activation by interfering with ASC oligomerization (13). This study advanced the understanding of quercetin from reducing inflammatory cytokine levels to regulating inflammasome assembly and IL-1 mediated vascular inflammation. The NLRP3/caspase-1/gasdermin D (GSDMD) axis controls the maturation and release of IL-1β and IL-18 and the occurrence of pyroptosis (43, 44). The regulatory effect of quercetin on this axis establishes a more direct mechanistic link with excessive innate immune activation, amplification of tissue injury, and sustained progression of chronic inflammation. The increasing recent proportion of disease-specific immune mechanism topics in LDA analysis is also consistent with the concentration of frontier keywords such as ferroptosis, pyroptosis, NLRP3, and IL-1β.

#### 4.3.4 Macrophage and microglial polarization and innate immune remodeling

A study by Tsai et al. published in Nutrients showed that quercetin downregulated M1 polarization related markers, including IL-6, TNF-α, IL-1β, C-C motif chemokine ligand 2 (CCL2), C-X-C motif chemokine ligand 10 (CXCL10), iNOS, and COX-2, while upregulating M2 polarization and antioxidant markers, including IL-10, CD206, HO-1, glutamate cysteine ligase catalytic subunit (GCLC), glutamate cysteine ligase modifier subunit (GCLM), and NAD(P)H quinone dehydrogenase 1 (NQO1) (45). This finding extends the role of quercetin from suppression of inflammatory cytokines to remodeling of innate immune cell functional states. Such cell state transitions are closely related to neuroinflammation, atherosclerosis, metabolic inflammation, and tissue repair. Microglia and macrophages can mediate pro-inflammatory injury during disease progression, but they also participate in debris clearance, tissue repair, and maintenance of immune homeostasis (46). The coordinated regulation of M1/M2 polarization, ROS generation, NO release, and antioxidant defense provides a cellular basis for the context dependent immune effects of quercetin across multiple disease models.

#### 4.3.5 Th17/Treg balance, mast cells, and adaptive immunity

The immunomodulatory activity of quercetin is not restricted to innate immunity. In a collagen induced arthritis model, Yan Yang et al. found that quercetin restored the Th17/Treg balance and downregulated pro-inflammatory molecules including NLRP3, caspase-1, IL-1β, TNF-α, IL-6, COX-2, and iNOS (14). This evidence links quercetin to autoimmune inflammation, T cell differentiation, and inflammasome regulation.

The field of allergic immunity also provides independent evidence for the relationship between quercetin and immune regulation. A study by Zuyi Weng et al. published in PLOS ONE showed that quercetin blocked cytokine release from human mast cells and exerted stronger regulatory effects than cromolyn on several indicators (15). Mast cells release histamine, prostaglandins, leukotrienes, and multiple cytokines, and they function as key effector cells in allergic inflammation and barrier tissue immune responses. This research direction supports the presence of allergy, mast cell activation, and immune mediated disease themes in the bibliometric analysis, and indicates that quercetin related research has extended from macrophage mediated inflammation toward adaptive immunity and allergic responses.

### 4.4 Latent topic identification based on LDA reveals disease research contexts

#### 4.4.1 Senescence, chronic inflammation, and immune remodeling

The LDA derived topic “senescence, aging and chronic inflammatory contexts” positioned quercetin within the research framework of immunosenescence, chronic low grade inflammation, and the senescence associated secretory phenotype (SASP). Senescent cells are not merely damaged cells in a quiescent state; they can continuously secrete IL-6, IL-8, TNF related factors, chemokines, matrix remodeling molecules, and profibrotic mediators, thereby driving inflammation within the tissue microenvironment, immune cell recruitment, and functional decline (47). This topic directly corresponded to keywords such as TNF, IL-6, oxidative stress, apoptosis, and senescence associated secretory phenotype, indicating that quercetin related immunological research has expanded from conventional anti inflammatory effects to the regulation of aging associated inflammatory networks.

Justice et al. conducted a first in human pilot study of dasatinib plus quercetin in patients with idiopathic pulmonary fibrosis, introducing quercetin into the research field of senolytics and senescent cell clearance (16). In a subsequent preliminary clinical study in participants with diabetic kidney disease, Hickson et al. reported that dasatinib plus quercetin reduced biological markers associated with senescent cell burden in humans (17). Although these studies used a combination regimen and therefore cannot attribute all observed effects to quercetin alone, they reframed the position of quercetin in immunological research. Quercetin is no longer considered only as a dietary flavonoid or a natural anti inflammatory molecule, but is increasingly incorporated into translational research frameworks involving senescent cell survival networks, SASP, tissue fibrosis, and chronic immune remodeling.

#### 4.4.2 Oxidative organ injury, metabolic inflammation, and oxidative immune coupling

The topic “systemic oxidative organ injury and metabolic stress” corresponded to the most stable oxidative stress axis in quercetin research. Immune injury in the liver, lung, kidney, cardiovascular system, and metabolic tissues commonly involves shared regulatory mechanisms, including excessive reactive oxygen species (ROS) generation, mitochondrial dysfunction, activation of the NF-κB pathway, insufficient Nrf2 mediated antioxidant defense, inflammatory cytokine release, and immune cell infiltration. This topic strongly overlapped with keywords such as oxidative stress, ROS, Nrf2, NF-κB, TNF, IL-6, and apoptosis, suggesting that oxidative organ injury represents a core disease context in quercetin immunity research.

Studies of lipopolysaccharide (LPS) induced acute lung injury have reported that quercetin can suppress ferroptosis through the Sirt1/Nrf2/Gpx4 axis and reduce pulmonary inflammation and oxidative damage (48). Studies using fine particulate matter (PM2.5) induced chronic lung injury models have also indicated that Nrf2 participates in ferroptosis regulation and that quercetin can alleviate pollutant exposure induced oxidative lung injury (49). In metabolic inflammation research, Overman et al. found in a coculture model of human macrophages and adipocytes that quercetin downregulated macrophage derived inflammatory gene expression and attenuated adipocyte inflammatory responses and insulin signaling dysfunction induced by macrophage conditioned medium (50). In research on obesity associated hypothalamic inflammation, Yang et al. further reported that quercetin attenuated microglia mediated inflammatory responses through heme oxygenase 1 (HO-1) induction (51).

Taken together, the regulation of immune function by quercetin is not limited to a single organ protective effect, but is mediated through crosstalk between redox homeostasis and immune inflammation. Pathological processes in metabolic tissues, lung tissue, and the central nervous system all involve ROS generation, NF-κB signaling activation, Nrf2 pathway regulation, macrophage or microglial activation, and sustained inflammatory cytokine release. By assigning these studies to the same latent topic, LDA further supports the interpretation that the disease application contexts of quercetin are grounded in a shared pathological basis of redox imbalance and amplified immune inflammation across multiple chronic diseases.

#### 4.4.3 Neuroinflammation, ferroptosis, and central immune injury

The topic “neuroinflammation, ferroptosis and brain injury” reflects the expansion of quercetin mediated immune regulation research into inflammatory disorders of the central nervous system. Microglia are the principal innate immune effector cells in the central nervous system, and their activation state is jointly regulated by ROS, NF-κB, mitogen activated protein kinase (MAPK), Nrf2, mitochondrial function, and lipid peroxidation. Neuroinflammatory processes involve not only the release of inflammatory cytokines such as TNF, IL-6, and IL-1β, but also neuronal injury, blood brain barrier disruption, mitochondrial stress, and inflammatory cell death.

Sun et al. reported in BV-2 microglial cells that quercetin attenuated LPS induced inflammatory responses and promoted Nrf2 pathway activation and HO-1 expression through MAPK related pathways (12). Kang et al. further showed that quercetin inhibited LPS induced nitric oxide (NO) production in BV-2 microglial cells, while suppressing the NF-κB pathway and activating the Nrf2 dependent HO-1 pathway (52). Studies using traumatic brain injury models have similarly reported that quercetin reduced brain edema, microglial activation, cortical inflammatory responses, and oxidative stress, with these effects being associated with activation of the Nrf2/HO-1 pathway (53).

The incorporation of ferroptosis has added a stronger frontier dimension to this topic. Ferroptosis is characterized by iron dependent lipid peroxidation and is closely related to neuronal injury, microglial activation, and oxidative inflammatory responses (54). Recent studies on cerebral ischemia and brain injury suggest that quercetin may suppress ferroptosis through the Nrf2/HO-1 signaling pathway, thereby reducing neural tissue injury (22). This mechanism links oxidative stress, Nrf2 signaling, ferroptosis, and neuroinflammatory disease into an integrated regulatory pathway, indicating that the research logic in neuroimmunology is shifting from simple reduction of inflammatory cytokines toward oxidative lipid injury, immune cell fate regulation, and maintenance of neural tissue homeostasis.

#### 4.4.4 Infection, antiviral responses, and intestinal mucosal immunity

The topic “infection, antiviral responses and intestinal inflammation” linked quercetin to pathogen recognition, viral replication, mucosal barrier function, and immunopathological injury. Infection induced immune responses require a dynamic balance between pathogen clearance and inflammatory tissue damage. Activation of the TLR/NF-κB pathway, ROS accumulation, inflammatory cytokine release, and excessive activation of the NLRP3/IL-1β axis can shift an otherwise protective immune response toward tissue injury. The research value of quercetin in this topic derives from its potential to regulate viral infection related processes, host inflammatory responses, and barrier tissue immune status simultaneously.

Modern studies have reported that quercetin can downregulate viral protein expression and reduce viral titers in small intestinal tissue, with its antiviral activity being associated with inhibition of early NF-κB pathway activation induced by rotavirus (55). Reviews and mechanistic studies related to coronavirus disease 2019 (COVID-19) have also incorporated quercetin into research frameworks concerning antiviral activity, immune regulation, and cytokine storm modulation (56).

Research on intestinal inflammation provides a clearer disease context for mechanisms related to mucosal immunity. Results from LPS induced intestinal inflammation models indicate that quercetin can inhibit inflammatory responses and pyroptosis through the TLR4/NF-κB/NLRP3 pathway (18). Further studies in ulcerative colitis models suggest that quercetin may influence intestinal mucosal homeostasis through multiple routes, including gut microbiota, tryptophan metabolism, aryl hydrocarbon receptor signaling, neutrophil extracellular traps, and tight junction barrier regulation (19, 20).

#### 4.4.5 Tumor associated immunity, cell fate regulation, and allergic inflammation

In the latent Dirichlet allocation topic model, the topics “cancer, apoptosis and intracellular signalling” and “allergy, mast cell activation and immune mediated disease” jointly indicate that quercetin related immune regulation research has extended into cell fate regulation, the tumor microenvironment, and allergic immunity. In cancer related literature, high frequency terms such as apoptosis, cell proliferation, NF-κB, PI3K/Akt, MAPK, STAT, and tumor microenvironment suggest that quercetin has been studied not only as a natural bioactive compound capable of directly inhibiting tumor cell proliferation and inducing tumor cell apoptosis, but also within the research context of the inflammatory tumor microenvironment and immune regulation.

Immunosuppressive cells, pro inflammatory cytokines, oxidative stress, and metabolic reprogramming within the tumor microenvironment can jointly regulate tumor progression and therapeutic response (57, 58). Existing reviews indicate that quercetin may modulate T cell infiltration, macrophage polarization, PD-L1 related signaling, the JAK/STAT pathway, and NF-κB mediated inflammatory responses (57). Breast cancer related studies further suggest that quercetin can interact with interferon γ (IFNγ) related signaling and regulate the expression of the IFNγ receptor, JAK2/STAT1, and PD-L1 (59).

Research on allergic responses and mast cells provides another line of cellular evidence for the immunomodulatory activity of quercetin. A study by Weng et al. published in PLOS ONE showed that quercetin inhibited cytokine release from human mast cells and reduced the release of inflammatory mediators, including histamine, IL-6, IL-8, TNF, tryptase, prostaglandin D2, and leukotrienes (15). Its inhibitory effects on several indicators were stronger than those of cromolyn, and the mechanism was associated with suppression of calcium influx and NF-κB activation.

#### 4.4.6 Delivery systems, bioavailability, and translational boundaries

The independent LDA topic “delivery, bioavailability and barrier system contexts” points to a core translational bottleneck in current quercetin immunity research. Free quercetin has poor aqueous solubility, low oral absorption efficiency, and rapid *in vivo* metabolism (24, 25). After oral intake, glucuronidated, sulfated, and methylated metabolites predominate in the circulation, making it difficult to maintain high concentrations of the parent compound (23, 60). Previous studies have shown that quercetin aglycone and its glycosides generally do not persist in plasma in large amounts in their original forms; instead, actual systemic exposure is mainly represented by conjugated metabolites (24, 25). This pharmacokinetic feature directly defines the interpretive boundary of quercetin related immune mechanisms.

Delivery research is transforming this limitation into an emerging research direction. Phospholipid complexation can substantially improve the aqueous solubility and oral absorption efficiency of quercetin (61). Strategies such as quercetin phytosomes, liposomes, polymer lipid hybrid nanoparticles, poly(lactic co glycolic acid) nanoparticles, and pH sensitive intestinal delivery systems are designed to improve quercetin stability, intestinal absorption, tissue distribution, or targeted release at inflammatory sites (62). Nanodelivery studies related to inflammatory bowel disease (IBD) have shown that quercetin loaded nanoparticles can improve quercetin stability and cellular uptake efficiency, while reducing disease severity, inflammatory cytokine expression, and intestinal barrier damage in dextran sulfate sodium (DSS) or oxazolone (OXA) induced colitis models (19).

#### 4.4.7 Mechanistic convergence among LDA-derived topics

The 12 LDA-derived topics are mechanistically interconnected rather than discrete. Oxidative organ injury, neuroinflammation, infection, intestinal inflammation and vascular fibrosis converge on a redox–inflammasome axis (32). Mitochondrial dysfunction and excessive reactive oxygen species promote NLRP3 priming and assembly, whereas Nrf2-dependent antioxidant responses, including the induction of HO-1 and NQO1, attenuate oxidative priming and restrain the caspase-1–IL-1β–GSDMD pathway (63). In turn, NLRP3 activation, cytokine release and pyroptotic tissue injury exacerbate mitochondrial dysfunction and reactive oxygen species generation (43). This crosstalk is context dependent, as Nrf2 has also been reported to facilitate inflammasome activation under specific experimental conditions (64). Accordingly, the reported effects of quercetin on Nrf2 activation and NLRP3 inhibition are better understood as coordinated modulation of a redox–inflammatory feedback circuit than as independent pharmacological actions (13, 63).

Senescence- and macrophage-related topics are similarly linked through reciprocal interactions between immune cells and tissue microenvironments. SASP mediators alter macrophage recruitment and function, whereas macrophage-derived inflammatory mediators can reinforce paracrine senescence (65). Macrophage-dependent clearance limits senescent-cell accumulation; excessive SASP can impair this process and establish a self-reinforcing inflammatory state (65). Macrophage-polarization signals identified in quercetin studies therefore intersect with senescence and chronic inflammation, although the binary M1/M2 framework does not capture the full continuum of macrophage states (46). The attenuation of pro-inflammatory macrophage programs by quercetin, together with its inclusion in dasatinib–quercetin senolytic regimens, places this compound at the interface between immune-cell state and senescent-cell burden (16, 17, 45). Nevertheless, direct evidence that quercetin concurrently modifies macrophage phenotype and SASP output within a single disease model remains limited. These interactions connect the LDA-derived topics through shared axes of redox regulation, inflammasome activity, immune-cell state, cellular senescence, tissue injury and exposure-dependent translation.

### 4.5 Systems pharmacology frontiers and multi-target mechanisms

Studies related to network pharmacology, molecular docking, systems pharmacology, protein–protein interaction, Kyoto Encyclopedia of Genes and Genomes (KEGG), and Gene Ontology (GO) were highly concentrated during 2021–2025, indicating that methodological approaches in this field are shifting from single-pathway validation toward systems-level mechanistic modeling. Quercetin is characterized by multi-target, multi-pathway, and multi-disease-context activity, and a research paradigm centered on a single receptor or pathway is insufficient to explain its full range of immunomodulatory effects (8). Systems pharmacology provides a methodological framework for integrating NF-κB, MAPK, PI3K/Akt, Nrf2, NLRP3, apoptosis, cytokine signaling pathways, and disease target networks (33–36, 66, 67).

The latent Dirichlet allocation (LDA) model is itself well suited for identifying the knowledge structure of this field. LDA is a generative probabilistic topic model in which each document is represented as a mixture of latent topics and each topic as a probability distribution over words (28). Compared with keyword co-occurrence analysis, LDA does not rely solely on discrete terms actively indexed by authors, but can extract latent semantic structures from combinations of titles, abstracts, and subject terms (28, 29). For quercetin-immunity research, an interdisciplinary field spanning natural products, pharmacology, immunology, disease models, and computational methods, LDA can reveal disease research contexts and methodological transitions that may be obscured by individual keywords (30).

### 4.6 Application barriers, future directions, and study limitations

The integration of three databases reduced coverage bias associated with reliance on a single database. Keyword co-occurrence analysis, co-citation analysis, burst analysis, thematic maturity assessment, and LDA topic modeling jointly characterized the explicit concepts, knowledge anchors, temporal evolution, and latent semantic structure of quercetin-immunity research. The analytical framework used in this study is suitable for mapping field-level developmental trajectories, but it cannot replace mechanistic experiments or clinical evidence. In addition, differences in database coverage, the restriction to English-language literature, incompleteness of bibliographic fields, variation in author-name standardization, heterogeneity in institutional address formats, and differences in citation accumulation time may have introduced bias into country-level, institution-level, author-level, and citation-based results.

Translational research needs to move beyond the broad question of anti-inflammatory activity and toward more precisely defined scientific problems. Future work should determine which form of quercetin or its metabolites acts on which immune-cell population, at which dose and exposure duration, during which disease stage, through which target network, and with which immunological outcome. Priority should be given to studies integrating single-cell omics, spatial transcriptomics, metabolomics, gut microbiota analysis, pharmacokinetic-pharmacodynamic modeling, and disease-specific immune models. Clinical research should further clarify formulation type, attainable systemic concentration, target-tissue exposure, immune endpoint measures, and safety boundaries, so that high-concentration *in vitro* effects are not directly extrapolated to humans.

### 4.7 Implications for quercetin-immunity research

The relationship between quercetin and immune regulation should be interpreted from a multilayered regulatory perspective. Chemical structure determines its redox activity and potential for protein interactions; metabolic transformation determines its *in vivo* exposure form; inflammatory signaling and oxidative stress define the mechanistic entry points of its core effects; immune-cell functional states determine its output in specific disease contexts; and delivery efficiency and bioavailability determine its translational feasibility. Keyword co-occurrence analysis revealed the core mechanistic axes of the field, whereas LDA analysis further clarified how these mechanisms are embedded in latent research themes such as aging, neuroinflammation, infection, intestinal inflammation, allergic immunity, and delivery optimization. The central direction of future research should not be repeated validation of broad anti-inflammatory activity, but the construction of an immunopharmacological evidence chain characterized by attainable dosing, defined targets, cell-type specificity, and disease-stage resolution.

## 5 Conclusion

From 2006 to 2025, immunological research on quercetin underwent a paradigm shift from screening the anti-inflammatory activity of a natural product to dissecting mechanisms within immune-regulatory networks. The 10,619 English-language articles and reviews obtained after deduplication across three bibliographic databases showed that the field has grown continuously over the past two decades and entered a rapid expansion phase during 2021–2025. The research focus has expanded from isolated pharmacodynamic observations to an interdisciplinary integration of inflammatory signaling pathways, oxidative stress, immune-cell functional regulation, disease-model construction, and systems pharmacology.

Keyword co-occurrence analysis constructed the explicit knowledge framework of the field. High-frequency core terms, including TNF, IL-6, IL-1β, NF-κB, ROS, Nrf2, apoptosis, COX-2, and iNOS, collectively pointed to five principal directions: pro-inflammatory cytokine regulatory networks, maintenance of redox homeostasis, transcription-factor regulation, inflammatory mediator release, and cell-fate determination. The immunopharmacological significance of quercetin should not be reduced to broad anti-inflammatory activity. Rather, it lies in multi-node regulation of inflammatory amplification, oxidative stress, signal transduction pathways, and immune-cell functional states.

Latent Dirichlet allocation (LDA) topic modeling complemented keyword analysis by revealing latent semantic structures that are difficult to capture using keyword frequency alone. Core topics, including inflammatory cytokines and signal transduction, systems pharmacology and computational mechanism analysis, senescence and chronic inflammation, oxidative organ injury, tumor-associated immune regulation, neuroinflammation, infection and intestinal inflammation, allergic responses and mast-cell activation, delivery systems, and bioavailability, clearly delineated the applied research contexts of quercetin immunology. The association between quercetin and immune regulation spans innate immunity, adaptive immunity, barrier immunity, neuroimmunity, metabolic inflammation, and aging-associated inflammation, rather than being confined to a single disease or pathway.

Over the past five years, research frontiers have concentrated on molecular docking, network pharmacology, systems pharmacology, protein–protein interaction, KEGG pathway enrichment analysis, GO functional enrichment analysis, Nrf2 signaling, ferroptosis, macrophage polarization, senescence-associated secretory phenotype, and RNA sequencing. The overall research paradigm is shifting from effect validation toward systematic mechanistic interpretation of the targets, pathways, cellular states, and disease networks through which quercetin exerts regulatory effects. The combined results of systems pharmacology analysis and LDA topic modeling indicate that the next phase of quercetin immunology should integrate computational prediction, mechanistic validation experiments, omics evidence, and pharmacokinetic characteristics.

The research value of quercetin derives from its bridging role between natural product chemistry and immunopathological regulatory networks. Its flavonol core structure confers molecular potential for redox regulation and protein interaction, whereas *in vivo* metabolic transformation determines its effective exposure form. The inflammatory signaling background and immune-cell functional state jointly shape its functional output in specific disease contexts. Future research should incorporate pharmacokinetic characteristics, active metabolite identification, single-cell omics, spatial omics, gut microbiota interactions, immune phenotyping, and clinically achievable dosing into a unified research framework. Only by clarifying *in vivo* exposure levels, molecular targets, immune-cell specificity, disease-stage dependence, and translational boundaries can quercetin advance from a highly studied natural flavonoid to an immunomodulatory candidate molecule with clearer mechanisms, more reliable evidence, and practical application potential.

## Data Availability

The original contributions presented in the study are included in the article/Supplementary material, further inquiries can be directed to the corresponding authors.

## References

[1] Kim JK Park SU. Quercetin and its role in biological functions: an updated review. EXCLI J. (2018) 17:856–63. doi: 10.17179/excli2018-1538, 30233284 PMC6141818

[2] Kuropatnicki AK Szliszka E Krol W. Historical aspects of propolis research in modern times. Evid Based Complement Alternat Med. (2013) 2013:964149. doi: 10.1155/2013/964149, 23710243 PMC3655583

[3] Rusznyák S Szent-Györgyi A. Vitamin P: flavonols as vitamins. Nature. (1936) 138:27–7. doi: 10.1038/138027a0

[4] Harborne JB Williams CA. Advances in flavonoid research since 1992. Phytochemistry. (2000) 55:481–504. doi: 10.1016/s0031-9422(00)00235-1, 11130659

[5] Manach C Scalbert A Morand C Rémésy C Jiménez L. Polyphenols: food sources and bioavailability. Am J Clin Nutr. (2004) 79:727–47. doi: 10.1093/ajcn/79.5.727 15113710

[6] Xu D Hu MJ Wang YQ Cui YL. Antioxidant activities of quercetin and its complexes for medicinal application. Molecules. (2019) 24:1123. doi: 10.3390/molecules24061123, 30901869 PMC6470739

[7] Boots AW Haenen GR Bast A. Health effects of quercetin: from antioxidant to nutraceutical. Eur J Pharmacol. (2008) 585:325–37. doi: 10.1016/j.ejphar.2008.03.008 18417116

[8] Li Y Yao J Han C Yang J Chaudhry MT Wang S . Quercetin, inflammation and immunity. Nutrients. (2016) 8:167. doi: 10.3390/nu8030167, 26999194 PMC4808895

[9] Medzhitov R. Origin and physiological roles of inflammation. Nature. (2008) 454:428–35. doi: 10.1038/nature07201, 18650913

[10] Nathan C Ding A. Nonresolving inflammation. Cell. (2010) 140:871–82. doi: 10.1016/j.cell.2010.02.029, 20303877

[11] Cho SY Park SJ Kwon MJ Jeong TS Bok SH Choi WY . Quercetin suppresses proinflammatory cytokines production through MAP kinases and NF-kappaB pathway in lipopolysaccharide-stimulated macrophage. Mol Cell Biochem. (2003) 243:153–60. doi: 10.1023/a:1021624520740, 12619901

[12] Sun GY Chen Z Jasmer KJ Chuang DY Gu Z Hannink M . Quercetin attenuates inflammatory responses in BV-2 microglial cells: role of MAPKs on the Nrf2 pathway and induction of Heme Oxygenase-1. PLoS One. (2015) 10:e0141509. doi: 10.1371/journal.pone.0141509, 26505893 PMC4624710

[13] Domiciano TP Wakita D Jones HD Crother TR Verri WA Jr Arditi M . Quercetin inhibits Inflammasome activation by interfering with ASC oligomerization and prevents Interleukin-1 mediated mouse Vasculitis. Sci Rep. (2017) 7:41539. doi: 10.1038/srep41539, 28148962 PMC5288648

[14] Yang Y Zhang X Xu M Wu X Zhao F Zhao C. Quercetin attenuates collagen-induced arthritis by restoration of Th17/Treg balance and activation of Heme oxygenase 1-mediated anti-inflammatory effect. Int Immunopharmacol. (2018) 54:153–62. doi: 10.1016/j.intimp.2017.11.013, 29149703

[15] Weng Z Zhang B Asadi S Sismanopoulos N Butcher A Fu X . Quercetin is more effective than cromolyn in blocking human mast cell cytokine release and inhibits contact dermatitis and photosensitivity in humans. PLoS One. (2012) 7:e33805. doi: 10.1371/journal.pone.0033805, 22470478 PMC3314669

[16] Justice JN Nambiar AM Tchkonia T LeBrasseur NK Pascual R Hashmi SK . Senolytics in idiopathic pulmonary fibrosis: results from a first-in-human, open-label, pilot study. EBioMedicine. (2019) 40:554–63. doi: 10.1016/j.ebiom.2018.12.052, 30616998 PMC6412088

[17] Hickson LJ Langhi Prata LGP Bobart SA Evans TK Giorgadze N Hashmi SK . Senolytics decrease senescent cells in humans: preliminary report from a clinical trial of Dasatinib plus quercetin in individuals with diabetic kidney disease. EBioMedicine. (2019) 47:446–56. doi: 10.1016/j.ebiom.2019.08.069, 31542391 PMC6796530

[18] Zhang HX Li YY Liu ZJ Wang JF. Quercetin effectively improves LPS-induced intestinal inflammation, pyroptosis, and disruption of the barrier function through the TLR4/NF-κB/NLRP3 signaling pathway *in vivo* and *in vitro*. Food Nutr Res. (2022) 66. doi: 10.29219/fnr.v66.8948, 36793340 PMC9899048

[19] Wang L Fu R Meng Y Liang J Xue W Hu H . pH sensitive quercetin nanoparticles ameliorate DSS-induced colitis in mice by Colon-specific delivery. Mol Nutr Food Res. (2024) 68:e2300051. doi: 10.1002/mnfr.202300051, 38010348

[20] Wei Q Jiang H Zeng J Xu J Zhang H Xiao E . Quercetin protected the gut barrier in ulcerative colitis by activating aryl hydrocarbon receptor. Phytomedicine. (2025) 140:156633. doi: 10.1016/j.phymed.2025.156633, 40088746

[21] Han X Xu T Fang Q Zhang H Yue L Hu G . Quercetin hinders microglial activation to alleviate neurotoxicity via the interplay between NLRP3 inflammasome and mitophagy. Redox Biol. (2021) 44:102010. doi: 10.1016/j.redox.2021.102010, 34082381 PMC8182123

[22] Peng C Ai Q Zhao F Li H Sun Y Tang K . Quercetin attenuates cerebral ischemic injury by inhibiting ferroptosis via Nrf2/HO-1 signaling pathway. Eur J Pharmacol. (2024) 963:176264. doi: 10.1016/j.ejphar.2023.176264, 38123006

[23] Manach C Williamson G Morand C Scalbert A Rémésy C. Bioavailability and bioefficacy of polyphenols in humans. I. Review of 97 bioavailability studies. Am J Clin Nutr. (2005) 81:230S–42S. doi: 10.1093/ajcn/81.1.230S, 15640486

[24] Zhu X Ding G Ren S Xi J Liu K. The bioavailability, absorption, metabolism, and regulation of glucolipid metabolism disorders by quercetin and its important glycosides: a review. Food Chem. (2024) 458:140262. doi: 10.1016/j.foodchem.2024.140262, 38944925

[25] Almeida AF Borge GIA Piskula M Tudose A Tudoreanu L Valentová K . Bioavailability of quercetin in humans with a focus on Interindividual variation. Compr Rev Food Sci Food Saf. (2018) 17:714–31. doi: 10.1111/1541-4337.12342, 33350133

[26] Donthu N Kumar S Mukherjee D Pandey N Lim WM. How to conduct a bibliometric analysis: an overview and guidelines. J Bus Res. (2021) 133:285–96. doi: 10.1016/j.jbusres.2021.04.070

[27] Aria M Cuccurullo C. Bibliometrix: an R-tool for comprehensive science mapping analysis. J Informetr. (2017) 11:959–75. doi: 10.1016/j.joi.2017.08.007

[28] Blei DM. Probabilistic topic models. Commun ACM. (2012) 55:77–84. doi: 10.1145/2133806.2133826

[29] Röder M. Both A. Hinneburg A. Exploring the space of topic coherence measures. Proceedings of the Eighth ACM International Conference on Web Search and Data Mining (2015) 399–408.

[30] Maier D Waldherr A Miltner P Wiedemann G Niekler A Keinert A . Applying LDA topic modeling in communication research: toward a valid and reliable methodology. Commun Methods Meas. (2018) 12:93–118. doi: 10.1080/19312458.2018.1430754

[31] Valko M Rhodes CJ Moncol J Izakovic M Mazur M. Free radicals, metals and antioxidants in oxidative stress-induced cancer. Chem Biol Interact. (2006) 160:1–40. doi: 10.1016/j.cbi.2005.12.009, 16430879

[32] Mangan MSJ Olhava EJ Roush WR Seidel HM Glick GD Latz E. Targeting the NLRP3 inflammasome in inflammatory diseases. Nat Rev Drug Discov. (2018) 17:588–606. doi: 10.1038/nrd.2018.97, 30026524

[33] Ru J Li P Wang J Zhou W Li B Huang C . TCMSP: a database of systems pharmacology for drug discovery from herbal medicines. J Cheminform. (2014) 6:13. doi: 10.1186/1758-2946-6-13, 24735618 PMC4001360

[34] Shannon P Markiel A Ozier O Baliga NS Wang JT Ramage D . Cytoscape: a software environment for integrated models of biomolecular interaction networks. Genome Res. (2003) 13:2498–504. doi: 10.1101/gr.1239303, 14597658 PMC403769

[35] Szklarczyk D Kirsch R Koutrouli M Nastou K Mehryary F Hachilif R . The STRING database in 2023: protein-protein association networks and functional enrichment analyses for any sequenced genome of interest. Nucleic Acids Res. (2023) 51:D638–46. doi: 10.1093/nar/gkac1000, 36370105 PMC9825434

[36] Trott O Olson AJ. AutoDock Vina: improving the speed and accuracy of docking with a new scoring function, efficient optimization, and multithreading. J Comput Chem. (2010) 31:455–61. doi: 10.1002/jcc.21334, 19499576 PMC3041641

[37] Endale M Park SC Kim S Kim SH Yang Y Cho JY . Quercetin disrupts tyrosine-phosphorylated phosphatidylinositol 3-kinase and myeloid differentiation factor-88 association, and inhibits MAPK/AP-1 and IKK/NF-κB-induced inflammatory mediators production in RAW 264.7 cells. Immunobiology. (2013) 218:1452–67. doi: 10.1016/j.imbio.2013.04.019, 23735482

[38] Kawai T Akira S. The role of pattern-recognition receptors in innate immunity: update on toll-like receptors. Nat Immunol. (2010) 11:373–84. doi: 10.1038/ni.1863, 20404851

[39] Karin M Ben-Neriah Y. Phosphorylation meets ubiquitination: the control of NF-[kappa]B activity. Annu Rev Immunol. (2000) 18:621–63. doi: 10.1146/annurev.immunol.18.1.621, 10837071

[40] Luo M Tian R Yang Z Peng YY Lu N. Quercetin suppressed NADPH oxidase-derived oxidative stress via heme oxygenase-1 induction in macrophages. Arch Biochem Biophys. (2019) 671:69–76. doi: 10.1016/j.abb.2019.06.007, 31251921

[41] Sul OJ Ra SW. Quercetin prevents LPS-induced oxidative stress and inflammation by modulating NOX2/ROS/NF-kB in lung epithelial cells. Molecules. (2021) 26:6949. doi: 10.3390/molecules26226949, 34834040 PMC8625571

[42] Kensler TW Wakabayashi N Biswal S. Cell survival responses to environmental stresses via the Keap1-Nrf2-ARE pathway. Annu Rev Pharmacol Toxicol. (2007) 47:89–116. doi: 10.1146/annurev.pharmtox.46.120604.141046, 16968214

[43] Broz P Dixit VM. Inflammasomes: mechanism of assembly, regulation and signalling. Nat Rev Immunol. (2016) 16:407–20. doi: 10.1038/nri.2016.58, 27291964

[44] Shi J Zhao Y Wang K Shi X Wang Y Huang H . Cleavage of GSDMD by inflammatory caspases determines pyroptotic cell death. Nature. (2015) 526:660–5. doi: 10.1038/nature15514, 26375003

[45] Tsai CF Chen GW Chen YC Shen CK Lu DY Yang LY . Regulatory effects of quercetin on M1/M2 macrophage polarization and oxidative/antioxidative balance. Nutrients. (2021) 14:67. doi: 10.3390/nu14010067, 35010945 PMC8746507

[46] Murray PJ Allen JE Biswas SK Fisher EA Gilroy DW Goerdt S . Macrophage activation and polarization: nomenclature and experimental guidelines. Immunity. (2014) 41:14–20. doi: 10.1016/j.immuni.2014.06.008, 25035950 PMC4123412

[47] Coppé JP Desprez PY Krtolica A Campisi J. The senescence-associated secretory phenotype: the dark side of tumor suppression. Annu Rev Pathol. (2010) 5:99–118. doi: 10.1146/annurev-pathol-121808-102144, 20078217 PMC4166495

[48] Deng S Li J Li L Lin S Yang Y Liu T . Quercetin alleviates lipopolysaccharide-induced acute lung injury by inhibiting ferroptosis via the Sirt1/Nrf2/Gpx4 pathway. Int J Mol Med. (2023) 52:118. doi: 10.3892/ijmm.2023.5321, 37888753 PMC10635686

[49] Ding S Jiang J Li Y. Quercetin alleviates PM2.5-induced chronic lung injury in mice by targeting ferroptosis. PeerJ. (2024) 12:e16703. doi: 10.7717/peerj.16703, 38188138 PMC10768656

[50] Overman A Chuang CC McIntosh M. Quercetin attenuates inflammation in human macrophages and adipocytes exposed to macrophage-conditioned media. Int J Obes. (2011) 35:1165–72. doi: 10.1038/ijo.2010.272, 21224828

[51] Yang J Kim CS Tu TH Tu T Kim M-S Goto T . Quercetin protects obesity-induced hypothalamic inflammation by reducing microglia-mediated inflammatory responses via HO-1 induction. Nutrients. (2017) 9:650. doi: 10.3390/nu9070650, 28644409 PMC5537770

[52] Kang CH Choi YH Moon SK Kim WJ Kim GY. Quercetin inhibits lipopolysaccharide-induced nitric oxide production in BV2 microglial cells by suppressing the NF-κB pathway and activating the Nrf2-dependent HO-1 pathway. Int Immunopharmacol. (2013) 17:808–13. doi: 10.1016/j.intimp.2013.09.009, 24076371

[53] Zhai X Wang Z Gao J. Quercetin alleviates microglial-induced inflammation after traumatic brain injury via the PGC-1α/Nrf2 pathway dependent on HDAC3 inhibition. Brain Res Bull. (2024) 217:111080. doi: 10.1016/j.brainresbull.2024.111080, 39277018

[54] Dixon SJ Lemberg KM Lamprecht MR Skouta R Zaitsev KM Gleason CE . Ferroptosis: an iron-dependent form of nonapoptotic cell death. Cell. (2012) 149:1060–72. doi: 10.1016/j.cell.2012.03.042, 22632970 PMC3367386

[55] Banerjee S Sarkar R Mukherjee A Miyoshi S Kitahara K Halder P . Quercetin, a flavonoid, combats rotavirus infection by deactivating rotavirus-induced pro-survival NF-κB pathway. Front Microbiol. (2022) 13:951716. doi: 10.3389/fmicb.2022.951716, 35983320 PMC9379144

[56] Colunga Biancatelli RML Berrill M Catravas JD Marik PE. Quercetin and vitamin C: an experimental, synergistic therapy for the prevention and treatment of SARS-CoV-2 related disease (COVID-19). Front Immunol. (2020) 11:1451. doi: 10.3389/fimmu.2020.01451, 32636851 PMC7318306

[57] Fang L Gao D Wang T Zhao H Zhang Y Wang S. From nature to clinic: quercetin's role in breast cancer immunomodulation. Front Immunol. (2024) 15:1483459. doi: 10.3389/fimmu.2024.1483459, 39712006 PMC11659267

[58] Li L Zhang M Liu T Li J Sun S Chen J . Quercetin-ferrum nanoparticles enhance photothermal therapy by modulating the tumor immunosuppressive microenvironment. Acta Biomater. (2022) 154:454–66. doi: 10.1016/j.actbio.2022.10.008, 36243377

[59] Qiu D Yan X Xiao X Zhang G Wang Y Cao J . To explore immune synergistic function of quercetin in inhibiting breast cancer cells. Cancer Cell Int. (2021) 21:632. doi: 10.1186/s12935-021-02345-5, 34838003 PMC8626953

[60] Di Pede G Bresciani L Calani L Petrangolini G Riva A Allegrini P . The human microbial metabolism of quercetin in different formulations: an in vitro evaluation. Foods. (2020) 9:1121. doi: 10.3390/foods9081121, 32823976 PMC7466208

[61] Riva A Ronchi M Petrangolini G Bosisio S Allegrini P. Improved Oral absorption of quercetin from quercetin Phytosome®, a new delivery system based on food grade lecithin. Eur J Drug Metab Pharmacokinet. (2019) 44:169–77. doi: 10.1007/s13318-018-0517-3, 30328058 PMC6418071

[62] Wang W Sun C Mao L Ma P Liu F Yang J . The biological activities, chemical stability, metabolism and delivery systems of quercetin: a review. Trends Food Sci Technol. (2016) 56:21–38. doi: 10.1016/j.tifs.2016.07.004

[63] Liu X Zhang X Ding Y Zhou W Tao L Lu P . Nuclear factor E2-related Factor-2 negatively regulates NLRP3 Inflammasome activity by inhibiting reactive oxygen species-induced NLRP3 priming. Antioxid Redox Signal. (2017) 26:28–43. doi: 10.1089/ars.2015.6615, 27308893 PMC5198158

[64] Garstkiewicz M Strittmatter GE Grossi S Sand J Fenini G Werner S . Opposing effects of Nrf2 and Nrf2-activating compounds on the NLRP3 inflammasome independent of Nrf2-mediated gene expression. Eur J Immunol. (2017) 47:806–17. doi: 10.1002/eji.201646665, 28247911

[65] Ogata Y Yamada T Hasegawa S Sanada A Iwata Y Arima M . SASP-induced macrophage dysfunction may contribute to accelerated senescent fibroblast accumulation in the dermis. Exp Dermatol. (2021) 30:84–91. doi: 10.1111/exd.14205, 33010063

[66] Kanehisa M Goto S. KEGG: Kyoto encyclopedia of genes and genomes. Nucleic Acids Res. (2000) 28:27–30. doi: 10.1093/nar/28.1.27, 10592173 PMC102409

[67] Ashburner M Ball CA Blake JA Botstein D Butler H Cherry JM . Gene ontology: tool for the unification of biology. The gene ontology consortium. Nat Genet. (2000) 25:25–9. doi: 10.1038/75556 PMC3037419 10802651

