# Supplementary material for: Knowledge structure, latent topics, and emerging trends in quercetin-immunity research from 2006 to 2025: a bibliometric and LDA topic-modeling analysis
Source: Front Nutr. 2026 Sep 9;13:1932000. doi: 10.3389/fnut.2026.1932000 (PMC13597670; doi:10.3389/fnut.2026.1932000)
Supplement: Supplementary file 1 [file Supplementary_file_1.docx]

**Supplementary Figures and Tables**

**Supplementary Figure 1.** Source-database overlap after cross-database deduplication.

**Supplementary Figure 2.** LDA model-selection diagnostics.

*The dashed vertical line marks the selected 12-topic model. K = 14 had a marginally higher score but produced more fragmented topics.*

**Supplementary Table 1.** Complete database-specific search strategies and screening records.

| **Database** | **Search field** | **Database-specific query** | **Time span** | **Document type** | **Language** | **Raw records** | **Eligible records** |
| --- | --- | --- | --- | --- | --- | --- | --- |
| Web of Science Core Collection | Topic | (quercetin OR quercetol) AND immun* | 2006-2025 | Article; Review | English | 3,680 | 3,598 |
| Scopus | Article title; Abstract; Keywords | (quercetin OR quercetol) AND immun* | 2006-2025 | Article; Review | English | 9,743 | 9,743 |
| PubMed | Title/Abstract | ((quercetin[Title/Abstract] OR quercetol[Title/Abstract]) AND immun*[Title/Abstract]) | 2006-2025 | Article; Review | English | 2,515 | 2,457 |

*Searches were conducted on 4 July 2026. In PubMed, the complete query additionally included (“2006/01/01”[Date - Publication] : “2025/12/31”[Date - Publication]) AND English[Language] AND (Journal Article[Publication Type] OR Review[Publication Type]); equivalent year, language, and document-type filters were applied in Web of Science Core Collection and Scopus.*

**Supplementary Table 2.** Complete keyword harmonization dictionary, including raw-to-canonical mappings and excluded terms

*Scope: author keywords, database-indexed keywords, and PubMed Medical Subject Headings from the 10,619-publication analytical dataset (2006–2025).*

| **Processing decision** | **Canonical label or exclusion class** | **Raw terms or variants** |
| --- | --- | --- |
| Mapped: abbreviation/spelling | AMPK | adenosine monophosphate activated protein kinase; Adenosine monophosphate-activated protein kinase; AMP-activated protein kinase; amp-activated protein kinase; AMPK; ampk; AMP‐activated protein kinase |
| Mapped: abbreviation/gene-protein name | COX-2 | COX-2; Cox-2; cox-2; COX2; cox2; Cyclooxygenase 2; cyclooxygenase 2; CYCLOOXYGENASE-2; Cyclooxygenase-2; cyclooxygenase-2; Cyclooxygenase2; prostaglandin endoperoxide synthase 2; prostaglandin-endoperoxide synthase 2 |
| Mapped: singular-plural/case | Gene Ontology | GENE ONTOLOGY; Gene Ontology; Gene ontology; gene ontology |
| Mapped: gene/protein name and spelling | HO-1 | Heme oxygenase 1; heme oxygenase 1; HEME OXYGENASE-1; Heme Oxygenase-1; Heme oxygenase-1; heme oxygenase-1; Heme-oxygenase-1; hemeoxygenase 1; Hemeoxygenase-1; HMOX1; HO-1; Ho-1; ho-1 |
| Mapped: abbreviation/symbol/hyphenation | IL-1β | IL-1 beta; IL-1-BETA; IL-1B; il-1b; IL-1beta; il-1beta; IL-1β; il-1β; IL1 beta; IL1β; il1β; IL‐1β; interleukin 1beta; Interleukin 1β; interleukin-1 beta; INTERLEUKIN-1-BETA; Interleukin-1beta; Interleukin-1β; interleukin-1β |
| Mapped: abbreviation/hyphenation | IL-6 | IL-6; Il-6; il-6; IL6; IL‐6; Interleukin - 6; interleukin - 6; Interleukin 6; interleukin 6; INTERLEUKIN-6; Interleukin-6; interleukin-6 |
| Mapped: abbreviation/full name | KEGG | KEGG; Kyoto Encyclopedia of Genes and Genomes |
| Mapped: abbreviation/singular-plural | LPS | LIPOPOLYSACCHARIDE; Lipopolysaccharide; lipopolysaccharide; Lipopolysaccharides; lipopolysaccharides; LPS; lps |
| Mapped: abbreviation/singular-plural | MAPK | MAPK; mapk; Mitogen Activated Protein Kinase; Mitogen activated protein kinase; mitogen activated protein kinase; Mitogen-activated protein kinase; mitogen-activated protein kinase; Mitogen-Activated Protein Kinases; Mitogen-activated protein kinases; mitogen-activated protein kinases |
| Mapped: abbreviation/symbol/spelling | NF-κB | immunoglobulin enhancer binding protein; NF kappa B; NF κB; NF- κ B; NF- κB; NF-kappa B; NF-kappa b; NF-KAPPA-B; NF-kappaB; nf-kappab; NF-KB; NF-kB; Nf-kb; nf-kb; NF-κ B; nf-κ b; NF-κB; NF-κb; Nf-κb; NFkB; nfkb; NFκ-B; NFκB; nfκb; NF‑κB; Nuclear Factor Kappa B; Nuclear factor kappa B; Nuclear factor kappa b; nuclear factor kappa B; Nuclear factor kappa-B; nuclear factor kappa-B; nuclear factor kappa-b; Nuclear factor kappaB; Nuclear factor κB; nuclear factor κB; nuclear factor κb; Nuclear Factor-Kappa B; Nuclear factor-kappa B; nuclear factor-kappa B; nuclear factor-kappa B transcription factor; NUCLEAR FACTOR-KAPPAB; Nuclear factor-kappaB; Nuclear factor-κB; nuclear factor-κB; nuclear factor-κb; Nuclear factor-κB transcription factor; Nuclear factorkappa B; Nuclearfactor-κB |
| Mapped: gene/protein name | NLRP3 | NLR Family Pyrin Domain Containing 3; nlr family pyrin domain containing 3; NLRP-3; NLRP3; nlrp3 |
| Mapped: database indexing/full-name form; gene/protein name and spelling; official gene symbol/protein display label | Nrf2 | NFE2L2; Nrf 2; Nrf-2; NRF2; Nrf2; nrf2; Nuclear factor (erythroid-derived 2)-like 2; nuclear factor (erythroid-derived 2)-like 2; Nuclear factor erythroid 2-related factor 2; nuclear factor erythroid 2-related factor 2; nuclear factor erythroid 2‑related factor 2; nuclear factor erythroid derived 2 like 2; Nuclear factor-erythroid 2-related factor-2; Nuclear factor-erythroid-2-related factor 2; transcription factor Nrf2 |
| Mapped: abbreviation/separator | PI3K/Akt | Phosphatidylinositol 3-kinase–akt; phosphatidylinositol-3-kinase/AKT; PI3K-AKT; PI3K-Akt; PI3K-AKT pathway; PI3K-Akt pathway; PI3K/AKT; PI3K/AkT; PI3K/Akt; pi3k/akt; PI3K/AKT pathway; PI3K/Akt pathway; PI3K/akt pathway; PI3K–Akt; pi3k–akt |
| Mapped: abbreviation/symbol/gene name | PPAR-γ | peroxisome proliferator activated receptor gamma; Peroxisome proliferator-activated receptor γ; Peroxisome proliferator-activated receptor-γ; peroxisome proliferator-activated receptor-γ; PPAR gamma; ppar gamma; PPAR-GAMMA; PPAR-γ; ppar-γ; PPARγ; pparγ |
| Mapped: abbreviation/synonym | ROS | reactive oxygen metabolite; Reactive Oxygen Species; Reactive oxygen species; reactive oxygen species; ROS; ros |
| Mapped: gene/protein name | SIRT1 | SIRT1; Sirt1; sirt1; SIRTUIN 1; Sirtuin 1; sirtuin 1; Sirtuin-1; Sirtuin1 |
| Mapped: abbreviation/hyphenation | TLR4 | TLR 4; tlr 4; TLR-4; tlr-4; TLR4; tlr4; toll like receptor 4; Toll-Like Receptor 4; Toll-like receptor 4; toll-like receptor 4 |
| Mapped: abbreviation/full name/Greek-letter form; abbreviation/letter form; abbreviation/spelling | TNF | (TNF)-α; (tnf)-α; TNF; tnf; TNF ALPHA; TNF α; TNF-; TNF- α; TNF-ALPHA; TNF-Alpha; TNF-alpha; tNF-alpha; tnf-alpha; TNF-α; Tnf-α; tnf-α; TNFa; TNFα; TNF‐α; Tumor necrosis factor; tumor necrosis factor; tumor necrosis factor al-pha; Tumor necrosis factor alpha; tumor necrosis factor alpha; Tumor Necrosis Factor-alpha; Tumor necrosis factor-alpha; tumor necrosis factor-alpha; Tumor Necrosis Factor-α; Tumor necrosis factor-α; tumor necrosis factor-α; TUMOR-NECROSIS-FACTOR; Tumour necrosis factor; tumour necrosis factor; Tumour necrosis factor α; Tumour necrosis factor-α |
| Mapped: hyphenation/singular-plural | anti-inflammatory activity | Anti-inflammatory activities; anti-inflammatory activities; Anti-Inflammatory activity; Anti-inflammatory Activity; Anti-inflammatory activity; anti-inflammatory activity; ANTIINFLAMMATORY ACTIVITIES; ANTIINFLAMMATORY ACTIVITY; Antiinflammatory activity; antiinflammatory activity |
| Mapped: hyphenation/singular-plural | anti-inflammatory agents | anti-inflammatory agent; Anti-Inflammatory Agents; Anti-inflammatory agents; anti-inflammatory agents; antiinflammatory agent; ANTIINFLAMMATORY AGENTS |
| Mapped: singular-plural | antioxidants | Anti-Oxidant; Anti-oxidant; anti-oxidant; Anti-oxidants; anti-oxidants; ANTIOXIDANT; Antioxidant; antioxidant; ANTIOXIDANTS; Antioxidants; antioxidants; antioxidant‌; anti⁃oxidant |
| Mapped: singular-plural | flavonoids | Flavonoid; flavonoid; FLAVONOIDS; Flavonoids; flavonoids; Flavono?i?ds |
| Mapped: abbreviation/gene-protein name | iNOS | i-NOS; Inducible Nitric Oxide Synthase; Inducible nitric oxide synthase; inducible nitric oxide synthase; INOS; iNOS; inos; NOS2; nos2 |
| Mapped: US/UK spelling | macrophage polarization | Macrophage polarisation; MACROPHAGE POLARIZATION; Macrophage polarization; macrophage polarization |
| Mapped: singular-plural | molecular docking | molecular dock-ing; MOLECULAR DOCKING; Molecular Docking; Molecular docking; molecular docking |
| Mapped: spelling/synonym | network pharmacology | Net-work pharmacology; network pharmacolo-gy; NETWORK PHARMACOLOGY; Network Pharmacology; Network pharmacology; network pharmacology |
| Mapped: singular-plural | plant extracts | Plant extract; plant extract; Plant Extracts; Plant extracts; plant extracts; PLANT-EXTRACTS |
| Mapped: hyphenation/singular-plural | protein-protein interaction | protein protein interaction; Protein-protein Interaction; Protein-protein interaction; protein-protein interaction; Protein-protein interactions; Protein‒protein interaction network; protein–protein interaction; protein–protein interactions; Protein−protein interactions |
| Excluded | Low-information indexing term | chemistry; down regulation; downregulation; drug effect; drug mechanism; drug therapy; metabolism; pathology; pharmacology; protein expression; unclassified drug; upregulation |
| Excluded | Model-system descriptor | animal cell; animal experiment; animal model; animal tissue; cell culture; cell line; cells cultured; human cell; in vitro study; in vivo study; mice; mouse; rat; rats; sprague dawley rat; wistar rat |
| Excluded | Population/indexing descriptor | adult; aged; animal; animals; female; human; humans; male; middle aged; non human; nonhuman; young adult |
| Excluded | Publication/indexing metadata | article; journal article; priority journal; review |
| Excluded | Routine laboratory method | enzyme linked immunosorbent assay; high performance liquid chromatography; immunohistochemistry; isolation and purification; real time polymerase chain reaction; western blotting |
| Excluded | Search-term artefact | 117-39-5; quercetin |
| Excluded | Study-design descriptor | comparative study; controlled study; dose response; dose response relationship drug; major clinical study; procedures; statistical analysis; statistics |

***Note.*** *Before dictionary lookup, terms underwent Unicode NFKC normalization, case-insensitive matching, whitespace normalization, and punctuation-insensitive comparison. Canonical labels retain the display capitalization, hyphenation, and Greek symbols shown above.*

***Abbreviations.*** *MeSH, Medical Subject Headings; PubMed, PubMed database; Scopus, Scopus database; WoSCC, Web of Science Core Collection.*

**Supplementary Table 3A**. Annual publication output by document type, 2006–2025.

| **Year** | **Total publications** | **Articles** | **Reviews** |
| --- | --- | --- | --- |
| 2006 | 101 | 59 | 42 |
| 2007 | 110 | 76 | 34 |
| 2008 | 110 | 83 | 27 |
| 2009 | 142 | 96 | 46 |
| 2010 | 198 | 141 | 57 |
| 2011 | 192 | 130 | 62 |
| 2012 | 239 | 182 | 57 |
| 2013 | 247 | 183 | 64 |
| 2014 | 267 | 217 | 50 |
| 2015 | 288 | 237 | 51 |
| 2016 | 333 | 262 | 71 |
| 2017 | 344 | 277 | 67 |
| 2018 | 385 | 300 | 85 |
| 2019 | 489 | 391 | 98 |
| 2020 | 734 | 520 | 214 |
| 2021 | 1019 | 638 | 381 |
| 2022 | 1202 | 792 | 410 |
| 2023 | 1165 | 737 | 428 |
| 2024 | 1373 | 1048 | 325 |
| 2025 | 1681 | 1385 | 296 |

**Supplementary Table 3B.** Stage-level collaboration indicators.

| **Period** | **Total publications** | **Articles** | **Reviews** | **Mean authors** | **Multi-country publications** | **Multi-country share (%)** | **Median citations** |
| --- | --- | --- | --- | --- | --- | --- | --- |
| 2021-2025 | 6440 | 4600 | 1840 | 6.97 | 1368 | 21.24 | 9.0 |
| 2016-2020 | 2285 | 1750 | 535 | 6.56 | 509 | 22.28 | 31.0 |
| 2011-2015 | 1233 | 949 | 284 | 5.47 | 241 | 19.55 | 40.0 |
| 2006-2010 | 661 | 455 | 206 | 5.08 | 113 | 17.10 | 54.0 |

**Supplementary Table 4.** Top countries/regions, institutions and author-name records.

**Countries/regions**

| **Rank** | **Name** | **Records** | **Share (%)** |
| --- | --- | --- | --- |
| 1 | China | 3877 | 36.51 |
| 2 | India | 1299 | 12.23 |
| 3 | United States | 1160 | 10.92 |
| 4 | South Korea | 573 | 5.40 |
| 5 | Italy | 472 | 4.44 |
| 6 | Egypt | 468 | 4.41 |
| 7 | Saudi Arabia | 386 | 3.63 |
| 8 | Iran | 384 | 3.62 |
| 9 | Brazil | 334 | 3.15 |
| 10 | Japan | 307 | 2.89 |
| 11 | United Kingdom | 300 | 2.83 |
| 12 | Germany | 264 | 2.49 |
| 13 | Turkey | 261 | 2.46 |
| 14 | Spain | 236 | 2.22 |
| 15 | Taiwan | 210 | 1.98 |
| 16 | Pakistan | 208 | 1.96 |
| 17 | Indonesia | 205 | 1.93 |
| 18 | Poland | 176 | 1.66 |
| 19 | Malaysia | 154 | 1.45 |
| 20 | Canada | 153 | 1.44 |

**Supplementary Table 4 continued.** Institutions

**Institutions**

| **Rank** | **Name** | **Records** | **Share (%)** |
| --- | --- | --- | --- |
| 1 | Beijing University of Chinese Medicine | 129 | 1.21 |
| 2 | Shanghai University of Traditional Chinese Medicine | 125 | 1.18 |
| 3 | Chengdu University of Traditional Chinese Medicine | 124 | 1.17 |
| 4 | Nanjing University of Chinese Medicine | 118 | 1.11 |
| 5 | Chinese Academy of Sciences | 114 | 1.07 |
| 6 | Cairo University | 110 | 1.04 |
| 7 | Guangzhou University of Chinese Medicine | 109 | 1.03 |
| 8 | King Saud University | 99 | 0.93 |
| 9 | China Academy of Chinese Medical Sciences | 87 | 0.82 |
| 10 | Shandong University of Traditional Chinese Medicine | 85 | 0.80 |
| 11 | Sichuan University | 80 | 0.75 |
| 12 | Zhejiang Chinese Medical University | 75 | 0.71 |
| 13 | Zagazig University | 73 | 0.69 |
| 14 | Huazhong University of Science and Technology | 71 | 0.67 |
| 15 | China Medical University | 68 | 0.64 |
| 16 | Southern Medical University | 67 | 0.63 |
| 17 | Tehran University of Medical Sciences | 65 | 0.61 |
| 18 | Zhejiang University | 63 | 0.59 |
| 19 | Central South University | 62 | 0.58 |
| 20 | Islamic Azad University | 59 | 0.56 |

**Supplementary Table 4 continued.** Author-name records

**Author-name records**

| **Rank** | **Name** | **Records** | **Share (%)** |
| --- | --- | --- | --- |
| 1 | Cho, Jae Youl | 38 | 0.36 |
| 2 | Wang, Wei | 34 | 0.32 |
| 3 | Zhang, Yi | 34 | 0.32 |
| 4 | Li, Jing | 29 | 0.27 |
| 5 | Li, Li | 27 | 0.25 |
| 6 | Wang, Jing | 26 | 0.24 |
| 7 | Zhang, Lei | 24 | 0.23 |
| 8 | Liu, Yang | 23 | 0.22 |
| 9 | Wang, Lei | 23 | 0.22 |
| 10 | Zhang, Wei | 23 | 0.22 |
| 11 | Wang, Yu | 22 | 0.21 |
| 12 | Wang, Yan | 22 | 0.21 |
| 13 | Zhang, Xin | 22 | 0.21 |
| 14 | Li, Yan | 22 | 0.21 |
| 15 | Liu, Ying | 21 | 0.20 |
| 16 | Zhang, Li | 21 | 0.20 |
| 17 | Wang, Yi | 20 | 0.19 |
| 18 | Kirkland, James L. | 20 | 0.19 |
| 19 | Zhang, Jing | 20 | 0.19 |
| 20 | Liu, Wei | 20 | 0.19 |

*Author-name records are based on standardised names and may include homonymous authors.*

**Supplementary Table 5A**. Leading harmonised keywords by publication-level frequency

| **Rank** | **Keyword** | **Records** | **Share (%)** | **Thematic category** |
| --- | --- | --- | --- | --- |
| 1 | flavonoids | 2,885 | 27.17 | Natural-product context |
| 2 | oxidative stress | 2,816 | 26.52 | Oxidative-stress axis |
| 3 | inflammation | 2,638 | 24.84 | Inflammatory regulation |
| 4 | signal transduction | 2,581 | 24.31 | Cell fate and signalling |
| 5 | TNF | 2,539 | 23.91 | Inflammatory regulation |
| 6 | apoptosis | 2,452 | 23.09 | Cell fate and signalling |
| 7 | NF-κB | 2,430 | 22.88 | Inflammatory regulation |
| 8 | antioxidants | 2,270 | 21.38 | Oxidative-stress axis |
| 9 | anti-inflammatory activity | 2,226 | 20.96 | Inflammatory regulation |
| 10 | antioxidant activity | 2,054 | 19.34 | Oxidative-stress axis |
| 11 | IL-6 | 2,037 | 19.18 | Inflammatory regulation |
| 12 | plant extracts | 2,036 | 19.17 | Natural-product context |
| 13 | kaempferol | 1,786 | 16.82 | Natural-product context |
| 14 | gene expression | 1,601 | 15.08 | Cell fate and signalling |
| 15 | IL-1β | 1,528 | 14.39 | Inflammatory regulation |
| 16 | ROS | 1,489 | 14.02 | Oxidative-stress axis |
| 17 | molecular docking | 1,439 | 13.55 | Systems pharmacology and methods |
| 18 | cell proliferation | 1,395 | 13.14 | Cell fate and signalling |
| 19 | resveratrol | 1,328 | 12.51 | Natural-product context |
| 20 | curcumin | 1,326 | 12.49 | Natural-product context |
| 21 | luteolin | 1,195 | 11.25 | Natural-product context |
| 22 | antineoplastic activity | 1,194 | 11.24 | Other |
| 23 | cell viability | 1,185 | 11.16 | Cell fate and signalling |
| 24 | anti-inflammatory agents | 1,167 | 10.99 | Other |
| 25 | genetics | 1,138 | 10.72 | Other |
| 26 | histopathology | 1,048 | 9.87 | Other |
| 27 | cytotoxicity | 1,045 | 9.84 | Other |
| 28 | enzyme activity | 1,004 | 9.45 | Other |
| 29 | herbaceous agent | 947 | 8.92 | Other |
| 30 | gallic acid | 943 | 8.88 | Other |

*Note.* Frequencies are publication-level counts after final keyword harmonisation; each canonical keyword was counted once per publication. Percentages use the 10,619 unique publications as the denominator.

**Supplementary Table 5B.** LDA topic summary.

| **Topic** | **LDA topic label** | **Mean topic weight (%)** | **Recent share (%)** | **Top weighted terms** |
| --- | --- | --- | --- | --- |
| 1 | Inflammatory cytokines and signalling | 8.27 | 45.0 | inflammatory, NF-kappaB, anti-inflammatory, inflammation, lps, TNF, activation, signaling, flavonoids, cytokines, produ… |
| 2 | Systems pharmacology and computational mechanisms | 8.90 | 87.9 | network pharmacology, chinese, active, molecular docking, signaling, medicine, network, identified, traditional, key, i… |
| 3 | Senescence, ageing and chronic inflammatory contexts | 17.69 | 63.5 | senescence, development, aging, cellular, chronic, patients, phytochemicals, evidence, new, current, molecular, cancer,… |
| 4 | Systemic oxidative organ injury and metabolic stress | 12.45 | 56.7 | liver, oxidative stress, increased, antioxidant, serum, blood, kidney, compared, damage, lipid, diet, tissue, days, inj… |
| 5 | Cancer, apoptosis and intracellular signalling | 8.72 | 53.3 | cancer, tumor, apoptosis, breast, growth, signaling, Akt, carcinoma, line, anticancer, proliferation, inhibition, cycle… |
| 6 | Bone-cartilage disease and cellular stress response | 3.05 | 57.7 | bone, heat, stem, stress, shock, hyperoside, gastric, hsp70, differentiation, taxifolin, tissue, regeneration, osteopor… |
| 7 | Vascular injury, fibrosis and wound repair | 6.87 | 57.1 | ros, endothelial, fibrosis, wound, lung, mitochondrial, vascular, assay, muscle, tissue, healing, activation, inflammat… |
| 8 | Oxidative stress and redox regulation | 11.39 | 52.7 | flavonoids, antioxidant, anti-inflammatory, phenolic, properties, leaves, chromatography, chemical, assay, leaf, phytoc… |
| 9 | Neuroinflammation, ferroptosis and brain injury | 5.61 | 62.5 | brain, injury, oxidative stress, Nrf2, neuroprotective, neuronal, apoptosis, ferroptosis, ischemia, neurons, neuroinfla… |
| 10 | Allergy, mast-cell activation and immune-mediated disease | 4.78 | 54.8 | binding, allergic, asthma, receptor, mast, molecular docking, flavonoids, inhibitors, silico, complex, mol, arthritis,… |
| 11 | Infection, antiviral responses and intestinal inflammation | 4.39 | 68.9 | COVID-19, infection, virus, viral, antiviral, SARS-CoV-2, respiratory, coronavirus, colitis, infections, hiv, replicati… |
| 12 | Delivery, bioavailability and barrier-system contexts | 7.88 | 67.5 | polyphenols, skin, delivery, nanoparticles, intestinal, curcumin, gut microbiota, properties, bioavailability, gut, eff… |

**Supplementary Table 5C.** Keywords with the strongest descriptive burst indices during 2021–2025

| **Keyword** | **Records in 2021–2025** | **Total records** | **Recent share (%)** | **Burst index** |
| --- | --- | --- | --- | --- |
| KEGG | 613 | 614 | 99.8 | 3.974 |
| systems pharmacology | 873 | 879 | 99.3 | 3.959 |
| ferroptosis | 136 | 136 | 100.0 | 3.914 |
| TNF signalling | 103 | 103 | 100.0 | 3.888 |
| M1 macrophage | 98 | 98 | 100.0 | 3.882 |
| Nrf2 signalling | 84 | 84 | 100.0 | 3.864 |
| M2 macrophage | 83 | 83 | 100.0 | 3.862 |
| senescence-associated secretory phenotype | 100 | 101 | 99.0 | 3.848 |
| network pharmacology | 788 | 826 | 95.4 | 3.802 |
| Gene Ontology | 607 | 644 | 94.3 | 3.753 |
| molecular docking | 1,311 | 1,439 | 91.1 | 3.637 |
| IL-1β | 1,161 | 1,528 | 76.0 | 3.034 |
| IL-6 | 1,516 | 2,037 | 74.4 | 2.973 |
| TNF | 1,729 | 2,539 | 68.1 | 2.721 |

*Note.* The burst index was calculated as (observed period frequency + 1)/(four-period mean expected frequency + 1). Terms with a total frequency below 20 or a period frequency below 10 were excluded from burst ranking.

**Supplementary Table 5D.** Theme coverage, internal density, recent share and strategic position

| **Theme** | **Records** | **Density proxy** | **Recent share** | **Strategic position** |
| --- | --- | --- | --- | --- |
| Inflammatory signalling and cytokine regulation | 6,480 | 430.47 | 64.1% | Motor theme |
| Natural products and comparative phytochemistry | 6,375 | 312.64 | 64.7% | Basic and transversal theme |
| Cell fate and signal transduction | 6,046 | 394.62 | 64.2% | Motor theme |
| Oxidative stress and redox homeostasis | 5,046 | 442.90 | 62.6% | Motor theme |
| Systems pharmacology and computational methods | 1,970 | 496.60 | 94.2% | Emerging methodological frontier |
| Emerging disease-specific immune mechanisms | 1,436 | 12.11 | 84.7% | Emerging or rapidly expanding theme |

*Note.* Records denote thematic coverage. The density proxy is the mean pairwise co-occurrence strength within each theme; recent share is the proportion of theme-keyword occurrences during 2021–2025.

**Supplementary Table 6.** Top 20 terms by component weight for each topic in the final 12-topic latent Dirichlet allocation model.

| **Topic** | **LDA topic label** | **Rank** | **Term** | **LDA component weight** |
| --- | --- | --- | --- | --- |
| 1 | Inflammatory cytokines and signalling | 1 | inflammatory | 3791.9581 |
| 1 | Inflammatory cytokines and signalling | 2 | NF-kappaB | 3779.2866 |
| 1 | Inflammatory cytokines and signalling | 3 | anti-inflammatory | 2806.9987 |
| 1 | Inflammatory cytokines and signalling | 4 | inflammation | 2606.5612 |
| 1 | Inflammatory cytokines and signalling | 5 | lps | 2175.9543 |
| 1 | Inflammatory cytokines and signalling | 6 | TNF | 1917.2129 |
| 1 | Inflammatory cytokines and signalling | 7 | activation | 1568.1076 |
| 1 | Inflammatory cytokines and signalling | 8 | signaling | 1515.6383 |
| 1 | Inflammatory cytokines and signalling | 9 | flavonoids | 1388.6305 |
| 1 | Inflammatory cytokines and signalling | 10 | cytokines | 1341.1281 |
| 1 | Inflammatory cytokines and signalling | 11 | production | 1340.4616 |
| 1 | Inflammatory cytokines and signalling | 12 | macrophages | 1075.8444 |
| 1 | Inflammatory cytokines and signalling | 13 | nuclear | 935.0098 |
| 1 | Inflammatory cytokines and signalling | 14 | MAPK | 919.2504 |
| 1 | Inflammatory cytokines and signalling | 15 | inhibited | 883.7546 |
| 1 | Inflammatory cytokines and signalling | 16 | iNOS | 842.2965 |
| 1 | Inflammatory cytokines and signalling | 17 | cytokine | 789.0723 |
| 1 | Inflammatory cytokines and signalling | 18 | stimulated | 752.0630 |
| 1 | Inflammatory cytokines and signalling | 19 | COX | 736.7922 |
| 1 | Inflammatory cytokines and signalling | 20 | mediated | 665.7681 |
| 2 | Systems pharmacology and computational mechanisms | 1 | network pharmacology | 2558.8661 |
| 2 | Systems pharmacology and computational mechanisms | 2 | chinese | 2463.1036 |
| 2 | Systems pharmacology and computational mechanisms | 3 | active | 2248.3474 |
| 2 | Systems pharmacology and computational mechanisms | 4 | molecular docking | 2224.8811 |
| 2 | Systems pharmacology and computational mechanisms | 5 | signaling | 1894.2332 |
| 2 | Systems pharmacology and computational mechanisms | 6 | medicine | 1742.6126 |
| 2 | Systems pharmacology and computational mechanisms | 7 | network | 1545.2152 |
| 2 | Systems pharmacology and computational mechanisms | 8 | identified | 1443.1868 |
| 2 | Systems pharmacology and computational mechanisms | 9 | traditional | 1398.1981 |
| 2 | Systems pharmacology and computational mechanisms | 10 | key | 1224.3724 |
| 2 | Systems pharmacology and computational mechanisms | 11 | ingredients | 1204.7674 |
| 2 | Systems pharmacology and computational mechanisms | 12 | KEGG | 1094.1980 |
| 2 | Systems pharmacology and computational mechanisms | 13 | database | 1042.3977 |
| 2 | Systems pharmacology and computational mechanisms | 14 | core | 1017.5029 |
| 2 | Systems pharmacology and computational mechanisms | 15 | enrichment | 1009.5788 |
| 2 | Systems pharmacology and computational mechanisms | 16 | decoction | 937.4896 |
| 2 | Systems pharmacology and computational mechanisms | 17 | Akt | 913.2187 |
| 2 | Systems pharmacology and computational mechanisms | 18 | PI3K | 836.2326 |
| 2 | Systems pharmacology and computational mechanisms | 19 | TNF | 789.3685 |
| 2 | Systems pharmacology and computational mechanisms | 20 | molecular | 788.3607 |
| 3 | Senescence, ageing and chronic inflammatory contexts | 1 | senescence | 1577.4391 |
| 3 | Senescence, ageing and chronic inflammatory contexts | 2 | development | 1341.0699 |
| 3 | Senescence, ageing and chronic inflammatory contexts | 3 | aging | 1228.1779 |
| 3 | Senescence, ageing and chronic inflammatory contexts | 4 | cellular | 1167.7110 |
| 3 | Senescence, ageing and chronic inflammatory contexts | 5 | chronic | 1069.3180 |
| 3 | Senescence, ageing and chronic inflammatory contexts | 6 | patients | 1051.6954 |
| 3 | Senescence, ageing and chronic inflammatory contexts | 7 | phytochemicals | 1019.2241 |
| 3 | Senescence, ageing and chronic inflammatory contexts | 8 | evidence | 956.2686 |
| 3 | Senescence, ageing and chronic inflammatory contexts | 9 | new | 946.9025 |
| 3 | Senescence, ageing and chronic inflammatory contexts | 10 | current | 906.9649 |
| 3 | Senescence, ageing and chronic inflammatory contexts | 11 | molecular | 862.9290 |
| 3 | Senescence, ageing and chronic inflammatory contexts | 12 | cancer | 836.2897 |
| 3 | Senescence, ageing and chronic inflammatory contexts | 13 | strategies | 816.7089 |
| 3 | Senescence, ageing and chronic inflammatory contexts | 14 | risk | 738.5414 |
| 3 | Senescence, ageing and chronic inflammatory contexts | 15 | inflammation | 737.1494 |
| 3 | Senescence, ageing and chronic inflammatory contexts | 16 | senescent | 733.7721 |
| 3 | Senescence, ageing and chronic inflammatory contexts | 17 | age | 712.6288 |
| 3 | Senescence, ageing and chronic inflammatory contexts | 18 | disorders | 707.1681 |
| 3 | Senescence, ageing and chronic inflammatory contexts | 19 | agents | 694.6378 |
| 3 | Senescence, ageing and chronic inflammatory contexts | 20 | dietary | 687.1162 |
| 4 | Systemic oxidative organ injury and metabolic stress | 1 | liver | 3309.6495 |
| 4 | Systemic oxidative organ injury and metabolic stress | 2 | oxidative stress | 1839.8814 |
| 4 | Systemic oxidative organ injury and metabolic stress | 3 | increased | 1562.6774 |
| 4 | Systemic oxidative organ injury and metabolic stress | 4 | antioxidant | 1443.1699 |
| 4 | Systemic oxidative organ injury and metabolic stress | 5 | serum | 1343.9407 |
| 4 | Systemic oxidative organ injury and metabolic stress | 6 | blood | 1287.4878 |
| 4 | Systemic oxidative organ injury and metabolic stress | 7 | kidney | 1163.3183 |
| 4 | Systemic oxidative organ injury and metabolic stress | 8 | compared | 1131.5322 |
| 4 | Systemic oxidative organ injury and metabolic stress | 9 | damage | 1128.7847 |
| 4 | Systemic oxidative organ injury and metabolic stress | 10 | lipid | 1116.9058 |
| 4 | Systemic oxidative organ injury and metabolic stress | 11 | diet | 1115.0090 |
| 4 | Systemic oxidative organ injury and metabolic stress | 12 | tissue | 1102.2032 |
| 4 | Systemic oxidative organ injury and metabolic stress | 13 | days | 1088.7719 |
| 4 | Systemic oxidative organ injury and metabolic stress | 14 | injury | 1058.4048 |
| 4 | Systemic oxidative organ injury and metabolic stress | 15 | weight | 1055.0632 |
| 4 | Systemic oxidative organ injury and metabolic stress | 16 | day | 1017.6521 |
| 4 | Systemic oxidative organ injury and metabolic stress | 17 | diabetic | 1011.8101 |
| 4 | Systemic oxidative organ injury and metabolic stress | 18 | decreased | 992.6714 |
| 4 | Systemic oxidative organ injury and metabolic stress | 19 | insulin | 972.7468 |
| 4 | Systemic oxidative organ injury and metabolic stress | 20 | reduced | 972.2615 |
| 5 | Cancer, apoptosis and intracellular signalling | 1 | cancer | 7621.3866 |
| 5 | Cancer, apoptosis and intracellular signalling | 2 | tumor | 2660.2055 |
| 5 | Cancer, apoptosis and intracellular signalling | 3 | apoptosis | 2466.4730 |
| 5 | Cancer, apoptosis and intracellular signalling | 4 | breast | 1316.8851 |
| 5 | Cancer, apoptosis and intracellular signalling | 5 | growth | 1105.4264 |
| 5 | Cancer, apoptosis and intracellular signalling | 6 | signaling | 1002.3512 |
| 5 | Cancer, apoptosis and intracellular signalling | 7 | Akt | 859.6757 |
| 5 | Cancer, apoptosis and intracellular signalling | 8 | carcinoma | 753.6355 |
| 5 | Cancer, apoptosis and intracellular signalling | 9 | line | 724.1804 |
| 5 | Cancer, apoptosis and intracellular signalling | 10 | anticancer | 717.8738 |
| 5 | Cancer, apoptosis and intracellular signalling | 11 | proliferation | 706.2551 |
| 5 | Cancer, apoptosis and intracellular signalling | 12 | inhibition | 693.6820 |
| 5 | Cancer, apoptosis and intracellular signalling | 13 | cycle | 675.3964 |
| 5 | Cancer, apoptosis and intracellular signalling | 14 | resistance | 629.4057 |
| 5 | Cancer, apoptosis and intracellular signalling | 15 | autophagy | 624.6198 |
| 5 | Cancer, apoptosis and intracellular signalling | 16 | survival | 611.6683 |
| 5 | Cancer, apoptosis and intracellular signalling | 17 | cell proliferation | 599.1280 |
| 5 | Cancer, apoptosis and intracellular signalling | 18 | lung | 591.0219 |
| 5 | Cancer, apoptosis and intracellular signalling | 19 | dna | 584.1675 |
| 5 | Cancer, apoptosis and intracellular signalling | 20 | assay | 576.8566 |
| 6 | Bone-cartilage disease and cellular stress response | 1 | bone | 1443.9243 |
| 6 | Bone-cartilage disease and cellular stress response | 2 | heat | 739.9759 |
| 6 | Bone-cartilage disease and cellular stress response | 3 | stem | 597.4403 |
| 6 | Bone-cartilage disease and cellular stress response | 4 | stress | 568.9295 |
| 6 | Bone-cartilage disease and cellular stress response | 5 | shock | 555.6203 |
| 6 | Bone-cartilage disease and cellular stress response | 6 | hyperoside | 484.2079 |
| 6 | Bone-cartilage disease and cellular stress response | 7 | gastric | 480.4942 |
| 6 | Bone-cartilage disease and cellular stress response | 8 | hsp70 | 476.9654 |
| 6 | Bone-cartilage disease and cellular stress response | 9 | differentiation | 417.8184 |
| 6 | Bone-cartilage disease and cellular stress response | 10 | taxifolin | 388.6279 |
| 6 | Bone-cartilage disease and cellular stress response | 11 | tissue | 387.2554 |
| 6 | Bone-cartilage disease and cellular stress response | 12 | regeneration | 319.5491 |
| 6 | Bone-cartilage disease and cellular stress response | 13 | osteoporosis | 302.0360 |
| 6 | Bone-cartilage disease and cellular stress response | 14 | osteoarthritis | 294.1877 |
| 6 | Bone-cartilage disease and cellular stress response | 15 | iron | 282.8520 |
| 6 | Bone-cartilage disease and cellular stress response | 16 | periodontitis | 270.8461 |
| 6 | Bone-cartilage disease and cellular stress response | 17 | marrow | 265.2922 |
| 6 | Bone-cartilage disease and cellular stress response | 18 | cartilage | 259.5099 |
| 6 | Bone-cartilage disease and cellular stress response | 19 | reticulum | 244.2027 |
| 6 | Bone-cartilage disease and cellular stress response | 20 | endoplasmic | 239.5639 |
| 7 | Vascular injury, fibrosis and wound repair | 1 | ros | 1036.8881 |
| 7 | Vascular injury, fibrosis and wound repair | 2 | endothelial | 1026.5730 |
| 7 | Vascular injury, fibrosis and wound repair | 3 | fibrosis | 980.8775 |
| 7 | Vascular injury, fibrosis and wound repair | 4 | wound | 843.0407 |
| 7 | Vascular injury, fibrosis and wound repair | 5 | lung | 838.2468 |
| 7 | Vascular injury, fibrosis and wound repair | 6 | mitochondrial | 815.7209 |
| 7 | Vascular injury, fibrosis and wound repair | 7 | vascular | 756.3063 |
| 7 | Vascular injury, fibrosis and wound repair | 8 | assay | 748.2144 |
| 7 | Vascular injury, fibrosis and wound repair | 9 | muscle | 664.5617 |
| 7 | Vascular injury, fibrosis and wound repair | 10 | tissue | 660.3074 |
| 7 | Vascular injury, fibrosis and wound repair | 11 | healing | 657.5405 |
| 7 | Vascular injury, fibrosis and wound repair | 12 | activation | 622.5191 |
| 7 | Vascular injury, fibrosis and wound repair | 13 | inflammation | 577.6932 |
| 7 | Vascular injury, fibrosis and wound repair | 14 | increased | 570.3374 |
| 7 | Vascular injury, fibrosis and wound repair | 15 | collagen | 546.9186 |
| 7 | Vascular injury, fibrosis and wound repair | 16 | reduced | 531.5908 |
| 7 | Vascular injury, fibrosis and wound repair | 17 | tgf | 527.7568 |
| 7 | Vascular injury, fibrosis and wound repair | 18 | pulmonary | 511.0614 |
| 7 | Vascular injury, fibrosis and wound repair | 19 | adhesion | 500.5661 |
| 7 | Vascular injury, fibrosis and wound repair | 20 | mmp | 496.6425 |
| 8 | Oxidative stress and redox regulation | 1 | flavonoids | 2810.7119 |
| 8 | Oxidative stress and redox regulation | 2 | antioxidant | 1697.2370 |
| 8 | Oxidative stress and redox regulation | 3 | anti-inflammatory | 1572.6672 |
| 8 | Oxidative stress and redox regulation | 4 | phenolic | 1200.7629 |
| 8 | Oxidative stress and redox regulation | 5 | properties | 1173.6319 |
| 8 | Oxidative stress and redox regulation | 6 | leaves | 1076.8133 |
| 8 | Oxidative stress and redox regulation | 7 | chromatography | 874.1653 |
| 8 | Oxidative stress and redox regulation | 8 | chemical | 787.1190 |
| 8 | Oxidative stress and redox regulation | 9 | assay | 781.3625 |
| 8 | Oxidative stress and redox regulation | 10 | leaf | 774.7150 |
| 8 | Oxidative stress and redox regulation | 11 | phytochemical | 771.3824 |
| 8 | Oxidative stress and redox regulation | 12 | high | 756.4161 |
| 8 | Oxidative stress and redox regulation | 13 | traditional | 745.4508 |
| 8 | Oxidative stress and redox regulation | 14 | total | 734.3667 |
| 8 | Oxidative stress and redox regulation | 15 | constituents | 717.1027 |
| 8 | Oxidative stress and redox regulation | 16 | antioxidant activity | 705.0977 |
| 8 | Oxidative stress and redox regulation | 17 | bioactive | 701.4298 |
| 8 | Oxidative stress and redox regulation | 18 | content | 696.0877 |
| 8 | Oxidative stress and redox regulation | 19 | isolated | 691.5618 |
| 8 | Oxidative stress and redox regulation | 20 | species | 682.4912 |
| 9 | Neuroinflammation, ferroptosis and brain injury | 1 | brain | 1838.0275 |
| 9 | Neuroinflammation, ferroptosis and brain injury | 2 | injury | 1026.7738 |
| 9 | Neuroinflammation, ferroptosis and brain injury | 3 | oxidative stress | 724.1046 |
| 9 | Neuroinflammation, ferroptosis and brain injury | 4 | Nrf2 | 700.6019 |
| 9 | Neuroinflammation, ferroptosis and brain injury | 5 | neuroprotective | 669.2793 |
| 9 | Neuroinflammation, ferroptosis and brain injury | 6 | neuronal | 567.0507 |
| 9 | Neuroinflammation, ferroptosis and brain injury | 7 | apoptosis | 536.6723 |
| 9 | Neuroinflammation, ferroptosis and brain injury | 8 | ferroptosis | 534.5395 |
| 9 | Neuroinflammation, ferroptosis and brain injury | 9 | ischemia | 533.9353 |
| 9 | Neuroinflammation, ferroptosis and brain injury | 10 | neurons | 475.1111 |
| 9 | Neuroinflammation, ferroptosis and brain injury | 11 | neuroinflammation | 472.6862 |
| 9 | Neuroinflammation, ferroptosis and brain injury | 12 | reperfusion | 463.6802 |
| 9 | Neuroinflammation, ferroptosis and brain injury | 13 | cognitive | 457.9286 |
| 9 | Neuroinflammation, ferroptosis and brain injury | 14 | neurodegenerative | 433.3116 |
| 9 | Neuroinflammation, ferroptosis and brain injury | 15 | damage | 422.9530 |
| 9 | Neuroinflammation, ferroptosis and brain injury | 16 | microglia | 390.6463 |
| 9 | Neuroinflammation, ferroptosis and brain injury | 17 | depression | 373.9551 |
| 9 | Neuroinflammation, ferroptosis and brain injury | 18 | cerebral | 370.3837 |
| 9 | Neuroinflammation, ferroptosis and brain injury | 19 | memory | 368.6121 |
| 9 | Neuroinflammation, ferroptosis and brain injury | 20 | stress | 362.8526 |
| 10 | Allergy, mast-cell activation and immune-mediated disease | 1 | binding | 1190.5549 |
| 10 | Allergy, mast-cell activation and immune-mediated disease | 2 | allergic | 805.2703 |
| 10 | Allergy, mast-cell activation and immune-mediated disease | 3 | asthma | 676.0532 |
| 10 | Allergy, mast-cell activation and immune-mediated disease | 4 | receptor | 596.3084 |
| 10 | Allergy, mast-cell activation and immune-mediated disease | 5 | mast | 543.8538 |
| 10 | Allergy, mast-cell activation and immune-mediated disease | 6 | molecular docking | 503.7184 |
| 10 | Allergy, mast-cell activation and immune-mediated disease | 7 | flavonoids | 439.2843 |
| 10 | Allergy, mast-cell activation and immune-mediated disease | 8 | inhibitors | 406.2721 |
| 10 | Allergy, mast-cell activation and immune-mediated disease | 9 | silico | 395.0174 |
| 10 | Allergy, mast-cell activation and immune-mediated disease | 10 | complex | 369.1264 |
| 10 | Allergy, mast-cell activation and immune-mediated disease | 11 | mol | 351.9734 |
| 10 | Allergy, mast-cell activation and immune-mediated disease | 12 | arthritis | 349.7895 |
| 10 | Allergy, mast-cell activation and immune-mediated disease | 13 | ige | 320.5257 |
| 10 | Allergy, mast-cell activation and immune-mediated disease | 14 | receptors | 320.2468 |
| 10 | Allergy, mast-cell activation and immune-mediated disease | 15 | interaction | 306.2567 |
| 10 | Allergy, mast-cell activation and immune-mediated disease | 16 | airway | 301.8497 |
| 10 | Allergy, mast-cell activation and immune-mediated disease | 17 | rheumatoid arthritis | 300.2266 |
| 10 | Allergy, mast-cell activation and immune-mediated disease | 18 | molecular | 295.5325 |
| 10 | Allergy, mast-cell activation and immune-mediated disease | 19 | enzyme | 292.7344 |
| 10 | Allergy, mast-cell activation and immune-mediated disease | 20 | structure | 291.9650 |
| 11 | Infection, antiviral responses and intestinal inflammation | 1 | COVID-19 | 1950.5763 |
| 11 | Infection, antiviral responses and intestinal inflammation | 2 | infection | 1677.8303 |
| 11 | Infection, antiviral responses and intestinal inflammation | 3 | virus | 1538.7861 |
| 11 | Infection, antiviral responses and intestinal inflammation | 4 | viral | 1156.4404 |
| 11 | Infection, antiviral responses and intestinal inflammation | 5 | antiviral | 1141.4912 |
| 11 | Infection, antiviral responses and intestinal inflammation | 6 | SARS-CoV-2 | 993.8129 |
| 11 | Infection, antiviral responses and intestinal inflammation | 7 | respiratory | 875.4667 |
| 11 | Infection, antiviral responses and intestinal inflammation | 8 | coronavirus | 762.2939 |
| 11 | Infection, antiviral responses and intestinal inflammation | 9 | colitis | 740.6820 |
| 11 | Infection, antiviral responses and intestinal inflammation | 10 | infections | 587.1028 |
| 11 | Infection, antiviral responses and intestinal inflammation | 11 | hiv | 486.7222 |
| 11 | Infection, antiviral responses and intestinal inflammation | 12 | replication | 450.0377 |
| 11 | Infection, antiviral responses and intestinal inflammation | 13 | host | 421.8404 |
| 11 | Infection, antiviral responses and intestinal inflammation | 14 | syndrome | 418.1356 |
| 11 | Infection, antiviral responses and intestinal inflammation | 15 | influenza | 394.1362 |
| 11 | Infection, antiviral responses and intestinal inflammation | 16 | ulcerative | 390.0762 |
| 11 | Infection, antiviral responses and intestinal inflammation | 17 | infected | 389.7500 |
| 11 | Infection, antiviral responses and intestinal inflammation | 18 | severe | 378.5524 |
| 11 | Infection, antiviral responses and intestinal inflammation | 19 | acute | 374.6500 |
| 11 | Infection, antiviral responses and intestinal inflammation | 20 | ibd | 349.6706 |
| 12 | Delivery, bioavailability and barrier-system contexts | 1 | polyphenols | 1719.9819 |
| 12 | Delivery, bioavailability and barrier-system contexts | 2 | skin | 1355.0654 |
| 12 | Delivery, bioavailability and barrier-system contexts | 3 | delivery | 1247.7764 |
| 12 | Delivery, bioavailability and barrier-system contexts | 4 | nanoparticles | 1011.9606 |
| 12 | Delivery, bioavailability and barrier-system contexts | 5 | intestinal | 865.3381 |
| 12 | Delivery, bioavailability and barrier-system contexts | 6 | curcumin | 674.7943 |
| 12 | Delivery, bioavailability and barrier-system contexts | 7 | gut microbiota | 610.4798 |
| 12 | Delivery, bioavailability and barrier-system contexts | 8 | properties | 585.6124 |
| 12 | Delivery, bioavailability and barrier-system contexts | 9 | bioavailability | 566.7818 |
| 12 | Delivery, bioavailability and barrier-system contexts | 10 | gut | 531.2006 |
| 12 | Delivery, bioavailability and barrier-system contexts | 11 | efficacy | 458.4648 |
| 12 | Delivery, bioavailability and barrier-system contexts | 12 | nps | 448.5090 |
| 12 | Delivery, bioavailability and barrier-system contexts | 13 | radiation | 427.9152 |
| 12 | Delivery, bioavailability and barrier-system contexts | 14 | bacterial | 427.8559 |
| 12 | Delivery, bioavailability and barrier-system contexts | 15 | systems | 418.1836 |
| 12 | Delivery, bioavailability and barrier-system contexts | 16 | antioxidant | 410.3158 |
| 12 | Delivery, bioavailability and barrier-system contexts | 17 | loaded | 404.8147 |
| 12 | Delivery, bioavailability and barrier-system contexts | 18 | release | 398.9674 |
| 12 | Delivery, bioavailability and barrier-system contexts | 19 | flavonoids | 385.5875 |
| 12 | Delivery, bioavailability and barrier-system contexts | 20 | tea | 360.2588 |

***Note:*** *Terms are ranked in descending order of their unnormalised component weights within each topic. Component weights were extracted from the topic-term matrix of the final model fitted to all 10,619 publications (K = 12); they are reported for within-topic ranking and should not be interpreted as posterior probabilities.*

**Supplementary Table 7.** Sensitivity analysis of country/region output under full and fractional counting.

| **Country/region** | **Full-count output** | **Fractional-count output** | **Full-count rank** | **Fractional-count rank** | **Rank change** | **Full-count inflation (%)** |
| --- | --- | --- | --- | --- | --- | --- |
| China | 3,877 | 3578.74 | 1 | 1 | 0 | 8.33 |
| India | 1,299 | 1090.10 | 2 | 2 | 0 | 19.16 |
| United States | 1,160 | 789.99 | 3 | 3 | 0 | 46.84 |
| South Korea | 573 | 488.40 | 4 | 4 | 0 | 17.32 |
| Italy | 472 | 346.01 | 5 | 5 | 0 | 36.41 |
| Egypt | 468 | 314.58 | 6 | 6 | 0 | 48.77 |
| Saudi Arabia | 386 | 177.03 | 7 | 11 | +4 | 118.05 |
| Iran | 384 | 299.81 | 8 | 7 | -1 | 28.08 |
| Brazil | 334 | 265.41 | 9 | 8 | -1 | 25.85 |
| Japan | 307 | 232.19 | 10 | 9 | -1 | 32.22 |
| United Kingdom | 300 | 163.09 | 11 | 13 | +2 | 83.94 |
| Germany | 264 | 159.39 | 12 | 14 | +2 | 65.63 |
| Turkey | 261 | 225.89 | 13 | 10 | -3 | 15.54 |
| Spain | 236 | 156.96 | 14 | 15 | +1 | 50.36 |
| Taiwan | 210 | 156.79 | 15 | 16 | +1 | 33.94 |
| Pakistan | 208 | 108.94 | 16 | 18 | +2 | 90.93 |
| Indonesia | 205 | 172.90 | 17 | 12 | -5 | 18.57 |
| Poland | 176 | 132.77 | 18 | 17 | -1 | 32.56 |
| Malaysia | 154 | 99.50 | 19 | 20 | +1 | 54.77 |
| Canada | 153 | 101.40 | 20 | 19 | -1 | 50.88 |

***Note****: Rows show the 20 leading countries/regions under full counting. For a publication involving m unique countries/regions, each represented country/region received a fractional weight of 1/m. Rank change was calculated as fractional-count rank minus full-count rank; positive values indicate a lower position under fractional counting. Full-count inflation was calculated as (full-count output/fractional-count output − 1) × 100%. The analysis included 10,535 publications with mapped country information; 84 publications without mapped country information were excluded. Full counting yielded 13,811 country credits, compared with 10,535 fractional credits, corresponding to an aggregate inflation of 31.10%. Rank concordance was high (Spearman ρ = 0.9904; Kendall τᵇ = 0.9346); overlap was 5/5 for the top five, 9/10 for the top 10, and 20/20 for the top 20 countries/regions.*

**Supplementary Table 8.** Sensitivity analysis of node- and edge-retention thresholds for the co-citation network.

| **Citation threshold, *C_i_*** | **Link threshold, *C_ij_*** | **Nodes, N** | **Edges, E** | **Density** | **Mean degree** | **Largest connected component, n (%)** | **Isolated nodes** |
| --- | --- | --- | --- | --- | --- | --- | --- |
| 10 | 3 | 851 | 5,623 | 0.0155 | 13.22 | 779 (91.54) | 70 |
| 10 | 5 | 851 | 1,531 | 0.0042 | 3.60 | 447 (52.53) | 374 |
| 10 | 10 | 851 | 182 | 0.0005 | 0.43 | 81 (9.52) | 726 |
| 15 | 3 | 397 | 3,371 | 0.0429 | 16.98 | 389 (97.98) | 8 |
| 15 | 5 | 397 | 1,112 | 0.0141 | 5.60 | 307 (77.33) | 88 |
| 15 | 10 | 397 | 173 | 0.0022 | 0.87 | 78 (19.65) | 282 |
| 20 | 3 | 241 | 2,222 | 0.0768 | 18.44 | 240 (99.59) | 1 |
| **20** | **5** | **241** | **822** | **0.0284** | **6.82** | **216 (89.63)** | **25** |
| 20 | 10 | 241 | 137 | 0.0047 | 1.14 | 64 (26.56) | 152 |
| 25 | 3 | 151 | 1,403 | 0.1239 | 18.58 | 151 (100.00) | 0 |
| 25 | 5 | 151 | 599 | 0.0529 | 7.93 | 144 (95.36) | 7 |
| 25 | 10 | 151 | 112 | 0.0099 | 1.48 | 55 (36.42) | 82 |
| 30 | 3 | 98 | 832 | 0.1750 | 16.98 | 98 (100.00) | 0 |
| 30 | 5 | 98 | 388 | 0.0816 | 7.92 | 97 (98.98) | 1 |
| 30 | 10 | 98 | 87 | 0.0183 | 1.78 | 44 (44.90) | 40 |
| 50 | 3 | 33 | 175 | 0.3314 | 10.61 | 33 (100.00) | 0 |
| 50 | 5 | 33 | 103 | 0.1951 | 6.24 | 29 (87.88) | 2 |
| 50 | 10 | 33 | 32 | 0.0606 | 1.94 | 18 (54.55) | 9 |

***Note.*** *Ci denotes the number of eligible Web of Science Core Collection source publications citing reference i; Cij denotes the number citing references i and j jointly. Density = 2E/[N(N - 1)], and mean degree = 2E/N. The bold row identifies the selected operational thresholds (Ci ≥ 20; Cij ≥ 5), which retained a large connected core without excessive network density. Percentages in the largest-connected-component column are calculated relative to the number of retained nodes.*

***Abbreviations.*** *E, number of retained co-citation links; N, number of retained reference nodes.*
